# Diagnostic and Prognostic Significance of Chemokines in Head and Neck Cancers: A Systematic Review and Meta-Analysis

**DOI:** 10.3390/cancers18091437

**Published:** 2026-04-30

**Authors:** Raneem Alsheikh, Deemah Assami, Dima Nasrallah, Ahmed Arabi, Ahmad Hamdan, Mohamed Ragab Elhadary, Ibrahim Elmakaty, Mohammed Imad Malki

**Affiliations:** 1 College of Medicine, QU Health, Qatar University, Doha P.O. Box 2713, Qatar; ra2104427@student.qu.edu.qa (R.A.); da2106263@qu.edu.qa (D.A.); dn2105571@qu.edu.qa (D.N.);; 2 Department of Medical Education, Hamad Medical Corporation, Doha P.O. Box 3050, Qatar; 3 Department of Basic Medical Sciences, College of Medicine, QU Health, Qatar University, Doha P.O. Box 2713, Qatar

**Keywords:** chemokines, biomarkers, head and neck cancers, diagnosis, prognosis, survival

## Abstract

Background: Head and neck cancers (HNCs) represent a global health burden, with high morbidity and mortality largely driven by late-stage diagnosis and heterogeneous clinical outcomes. Reliable biomarkers are needed to improve early detection, prognostic stratification, and therapeutic decision-making. This study evaluates the diagnostic and prognostic value of chemokines in HNCs and identifies candidates with clinical relevance. Methods: A comprehensive literature search was conducted on 28 March 2026 across PubMed, Embase, Scopus, Web of Science, and EBSCO. Observational studies involving patients or biological samples with confirmed HNCs were included if they evaluated chemokines as diagnostic or prognostic biomarkers. Risk of bias was assessed using the Newcastle–Ottawa Scale (NOS) for prognostic studies and the Quality Assessment of Diagnostic Accuracy Studies-2 (QUADAS-2) tool for diagnostic studies. Meta-analyses were performed for chemokines evaluated in ≥3 studies using the inverse-variance heterogeneity model. Results: Forty-four studies encompassing 7294 participants were included. Prognostic findings were heterogeneous across biomarkers. MIP-3α demonstrated consistently significant associations with poorer survival outcomes. In contrast, IL-8, CXCL10, and CXCR4 showed inconsistent or predominantly non-significant associations with overall survival (OS), disease-free survival (DFS), and locoregional control (LRC). For diagnostic performance, IL-8, in saliva, demonstrated relatively high sensitivity and specificity for oral squamous cell carcinoma. Chemerin showed high diagnostic accuracy, although evidence remains limited. Conclusions: Certain chemokines show potential as diagnostic and prognostic biomarkers in HNCs. However, heterogeneity across tumor types, samples, and methodologies limits evidence. Larger, well-designed studies are needed to validate their clinical utility in risk stratification and personalized management of HNCs.

## Simple Summary

Head and neck cancers are a major cause of illness and death worldwide, largely because they are often diagnosed late and their clinical course can vary widely between patients. There is a growing need for reliable biological markers that can help doctors detect these cancers earlier and better predict patient outcomes. Chemokines are small signaling proteins that play an important role in inflammation and immune responses and may also influence cancer development and progression. In this study, we evaluated existing studies to understand whether chemokines can be used to support the diagnosis of head and neck cancers or to estimate survival outcomes. Our findings show that certain chemokines are strongly associated with poor prognosis, while others demonstrate potential value in cancer detection. This work highlights the clinical relevance of chemokines and supports further research into their use as diagnostic tools, prognostic markers, and possible therapeutic targets in head and neck cancers.

## 1. Introduction

Head and neck cancers (HNCs) are among the most prevalent malignancies worldwide, ranking as the sixth most common type of cancer with more than 940,000 new cases and more than 480,000 deaths reported annually [1]. HNCs pose a multifaceted clinical challenge due to their diverse etiological factors, including human papillomavirus (HPV) infection, tobacco use, and alcohol consumption [2]. The progressive nature of HNCs often results in late-stage diagnosis, limiting treatment options and reducing survival chances [3,4]. Furthermore, conventional prognostic methods, which involve clinical staging and tissue biopsy, often fail to capture the complex molecular and cellular interplay within the tumor microenvironment, limiting their predictive ability [5]. This highlights the pressing need to develop reliable biomarkers to advance the diagnostic and prognostic accuracy [6], especially in early-stage HNCs. Among the various molecular candidates, chemokines have emerged as promising biomarkers in cancer research [7]. Chemokines are small cytokines that play an essential role in immune surveillance by regulating the migration of immune cells to sites of inflammation and injury through binding to G-protein-coupled receptors on the surface of target cells [8,9]. In the context of cancer, chemokines have been recognized as critical modulators of tumor biology, influencing processes such as tumor growth, metastasis, and the immune response within the tumor microenvironment [10,11]. Specifically, in HNCs, chemokines recruit immune cells like macrophages and lymphocytes to the tumor site, thereby shaping both local and systemic immune responses [12,13]. Chemokines such as CXCL12 and CCL2 have been implicated in promoting tumor invasion and metastasis by fostering angiogenesis, epithelial-to-mesenchymal transition (EMT), and immune evasion, making them key players in cancer progression [14,15]. Given their integral role in cancer development, chemokines hold significant potential as biomarkers for both diagnostic and prognostic purposes. Biomarkers are biological molecules that provide real-time data on physiological states, and in the case of cancer, chemokines could offer valuable insights into tumor aggressiveness, immune infiltration, and treatment response [16,17]. Aberrant chemokine levels in the tumor microenvironment or circulation could serve as indicators of disease status, facilitating earlier detection and more accurate predictions of recurrence or metastasis. By measuring chemokine levels, clinicians could gain a more nuanced understanding of a patient’s cancer, surpassing the limitations of traditional diagnostic and prognostic methods.

Although numerous primary studies have investigated chemokines in HNCs, their clinical utility remains uncertain due to inconsistent and heterogeneous findings. Variations in tumor subtype, biological specimen source, analytical techniques, and reported clinical outcomes contribute to differences in observed diagnostic and prognostic performance across studies. Moreover, the context-dependent nature of chemokine signaling within the tumor microenvironment, influenced by tumor biology and etiological factors such as HPV status, further complicates interpretation and limits comparability across studies [15,18]. Collectively, these challenges highlight the need for a structured synthesis of the available evidence to clarify the clinical relevance of chemokines in HNCs. Consequently, while previous reviews have broadly examined inflammatory mediators in HNCs, chemokines have often been evaluated collectively with other cytokines, without focused assessment of their distinct biological roles in immune cell trafficking, tumor progression, and metastasis. Moreover, the combined diagnostic and prognostic relevance of chemokines across different HNC subtypes and specimen types has not been comprehensively synthesized. Therefore, a focused and integrative evaluation of chemokines is needed to clarify their potential clinical relevance. This systematic review aims to synthesize the available evidence on chemokines as diagnostic and prognostic biomarkers in HNCs, while identifying key sources of heterogeneity that may explain inconsistencies in the literature and guide future research toward clinically translatable biomarker strategies.

## 2. Materials and Methods

### 2.1. Study Design and Registration

Our systematic review of observational studies was conducted following the Preferred Reporting Items for Systematic Reviews and Meta-Analyses (PRISMA) guidelines [19]. The protocol of this study is registered in the International Prospective Register of Systematic Reviews (PROSPERO) (PROSPERO ID: CRD42024585542). The PRISMA checklist is available in the Supplementary Material (Table S1).

### 2.2. Search Strategy

We performed our search strategy in multiple electronic databases, including PubMed, Embase, Scopus, Web of Science, and EBSCO. The search was conducted on 5 June 2024 without restrictions on the date or language to ensure comprehensive retrieval of studies. The search was then updated on 28 March 2026. Our search strategy was initially developed for PubMed and included multiple Medical Subject Heading (MeSH) terms such as ‘Chemokines’[MeSH], ‘Biomarkers’[MeSH], ‘Prognosis’[MeSH], ‘Diagnosis’[MeSH], ‘Head and Neck Neoplasms’[MeSH], ‘Oropharyngeal Neoplasms’[MeSH], and ‘Laryngeal Neoplasms’[MeSH], along with relevant keywords related to chemokines, biomarkers, prognosis, diagnosis, and HNCs. The search strategy was then transferred to the remaining databases using the Polyglot translator [20]. The full search strategy for all databases can be found in the Supplementary Material (Table S2).

### 2.3. Eligibility

Studies were included if they were original articles that used any observational study designs and involved patients or samples with confirmed HNCs. Eligible studies examined the role of chemokines as biomarkers, with a focus on their prognostic or diagnostic use in HNCs. Reviews, non-original articles, or non-peer-reviewed articles, including opinion pieces and editorials, were excluded. Studies involving animals or in vitro research without human subjects were excluded, as well as those not involving patients with HNCs or where mixed cancer populations were analyzed without separate data for HNCs. Studies not related to chemokines as biomarkers, or those that failed to report relevant diagnostic or prognostic outcomes, were also excluded. In cases where multiple studies used duplicate or similar datasets, only the most recent study was included. Studies published in languages other than English were also excluded due to resource limitations.

### 2.4. Study Selection and Screening

The articles retrieved from the database searches were imported into EndNote software 2025 for duplicate removal. Following this, the studies were transferred to Rayyan [21], a systematic review management tool, to identify any additional duplicates. Blinding was ensured during the study selection process, with title and abstract screening performed by two independent reviewers. Full-text screening was similarly conducted on the selected articles to determine the final set for inclusion in the analysis. Reasons for article exclusion were recorded. In cases of disagreement in these screening processes, a third reviewer was consulted to reach a consensus.

### 2.5. Data Extraction

Data extraction was performed by three pairs of two independent authors (RA, DA, DN, AA, AH, MRE) using Microsoft Excel 2021 with disagreements resolved by the senior author (MIM). The extracted data included the overall baseline characteristics, such as authors’ names, year of publication, sample size, mean/median age of the sample, cancer type, biomarker of interest, sample source, and detection technique. It also involved different diagnostic indices including true positives (TP), true negatives (TN), false positives (FP), false negative (FN), sensitivity and specificity. HRs of overall survival (OS), disease-free survival (DFS), distant metastasis-free survival (DMFS), disease-specific survival (DSS), metastasis-free survival (MFS), cancer-specific survival (CSS), progression-free survival (PFS), recurrence-free survival (RFS), and loco-regional control (LRC), were all reported for prognostic performance assessment. For studies presenting Kaplan–Meier curves without HRs, WebPlotDigitizer was used to extrapolate the curves, and the *ipdfc* module in Stata was used to estimate HRs [22,23]. If a single study reported multiple biomarkers or sample sources, each combination of biomarker and sample source was treated as a separate dataset for extraction and analysis.

### 2.6. Risk of Bias Assessment

The risk of bias for the included non-diagnostic studies was assessed using the Newcastle–Ottawa Scale (NOS) [24]. This tool is used for evaluating the methodological risk of bias of non-randomized studies. It assesses studies based on three main criteria: selection of study groups, comparability of groups, and ascertainment of the outcome or exposure of interest. Each category is further subdivided into specific items, with a star system used to assess risk of bias quality. A higher number of stars reflects better methodological quality, allowing for a more standardized assessment of study bias. Included diagnostic studies were evaluated using the Quality Assessment of Diagnostic Accuracy Studies-2 (QUADAS-2) scale [25]. This tool is designed to assess the quality of diagnostic accuracy studies by examining four key domains: patient selection, index test, reference standard, and flow and timing. Each domain is evaluated for risk of bias, with additional consideration of applicability in the first three domains. Questions in each of these four domains were treated as safeguards against bias and assigned “1” if implemented or “0” if not implemented or not mentioned.

### 2.7. Outcomes Definition

In this research, we assessed multiple oncologic outcomes to evaluate the prognostic role of chemokines in head and neck cancer. OS is defined as the time from diagnosis or treatment initiation to death from any cause. DFS refers to the time after curative treatment during which no signs or symptoms of cancer are present. DMFS is the time from treatment to the development of distant metastases. DSS and CSS represent the time from diagnosis to death specifically due to cancer, excluding other causes. MFS is the time to the occurrence of any metastasis, whether regional or distant. PFS is defined as the time from treatment initiation to disease progression or death from any cause. RFS is the time from treatment to cancer recurrence at any site. LRC refers to the duration without tumor recurrence in the primary site or regional lymph nodes.

### 2.8. Data Synthesis

The extracted data from each article were compiled and organized into tables. These findings were then described narratively in the text to emphasize key results and identify the most relevant chemokines. Descriptive statistics were used to analyze and present the data. Continuous variables were reported as means, while dichotomous variables were summarized using frequencies and percentages. For prognostic outcomes with more than three studies evaluating the same chemokine, a meta-analysis was conducted to generate summary forest plots using the inverse-variance heterogeneity model to account for the small number of studies and heterogeneity [1,26]. Heterogeneity between studies was assessed using the I^2^ statistic [27], while publication bias was evaluated using Doi plots and the LFK index due to the small number of studies included in each quantitative synthesis [28]. All analyses were performed using MetaXL version 5.3 (EpiGear Int Pty Ltd., Sunrise Beach, Australia) [29]. No subgroup analyses or sensitivity analyses were possible due to the limited number of studies in each synthesis.

## 3. Results

### 3.1. Study Selection Process 

**Figure 1:**
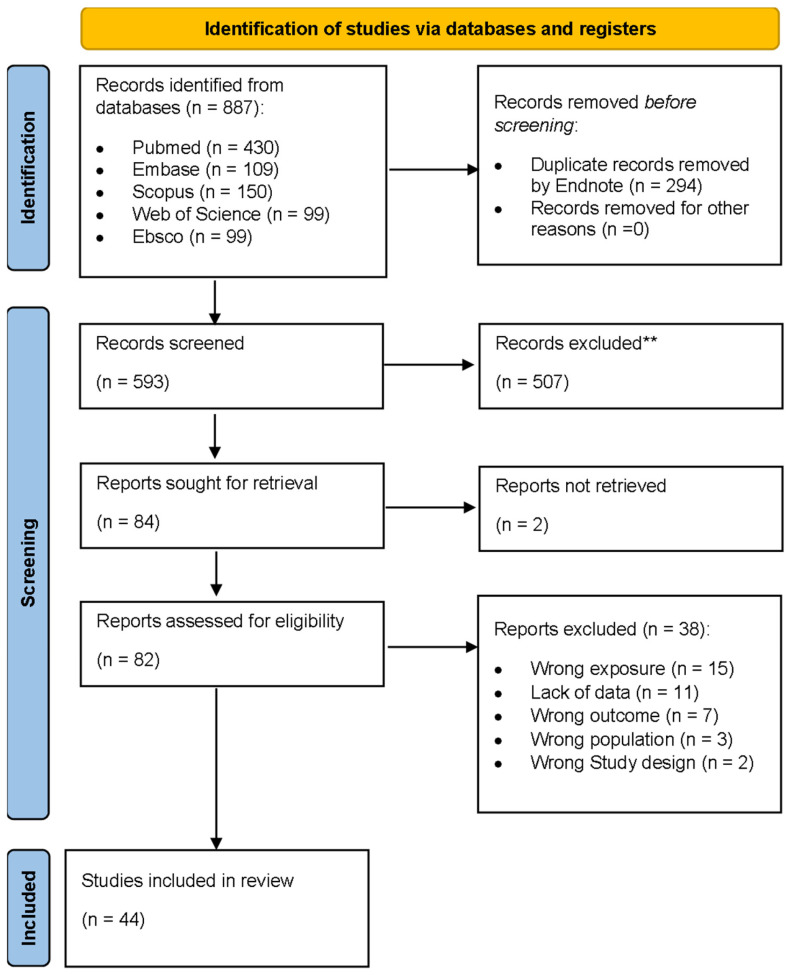
Literature search flow diagram for this systematic review. ** number of excluded papers.

Figure 1 shows the PRISMA flow diagram illustrating the study selection process. Our database search initially yielded 887 studies. After removing 294 duplicates using EndNote and Rayyan, 593 studies proceeded to title and abstract screening. From those, 84 papers were deemed eligible for full text screening. However, two papers were excluded as we were unable to retrieve their full text. The remaining 82 papers underwent full-text screening, resulting in the exclusion of 38 studies for reasons that include wrong exposure, lack of data, wrong outcome, wrong population, and wrong study design (Figure 1). Ultimately, 44 studies were included in this systematic review [25,26,27,28,29,30,31,32,33,34,35,36,37,38,39,40,41,42,43,44,45,46,47,48,49,50,51,52,53,54,55,56,57,58,59,60,61,62,63,64].

### 3.2. Study Characteristics and Patients’ Demographics

Table 1 provides a comprehensive summary of the characteristics of the included studies. A total of 44 articles, collectively reported data on 7294 participants from 17 different countries, with most participants being male. All studies were published in English, with China emerging as the country with the highest representation, contributing 16 (36.4%) studies. The most investigated condition was oral squamous cell carcinoma (OSCC) featured in 21 (47.7%) studies, followed by nasopharyngeal carcinoma (NPC), with a total of 10 (22.7%) studies. The sample type analyzed varied between salivary, serum and tissue samples, with tissue samples being the most frequently used (20 studies, 45.5%). Overall, the included studies were predominantly tissue-based investigations of OSCC and largely conducted in China.

### 3.3. Prognostic Performance of Chemokines

Of the studies included in this systematic review, 32 papers investigated the prognostic performance of various biomarkers. The most frequently reported biomarkers, defined as those investigated in three or more studies, are presented in the results section. The remaining biomarkers can be found in Table S3.

#### 3.3.1. Serum IL-8

**Figure 2:**
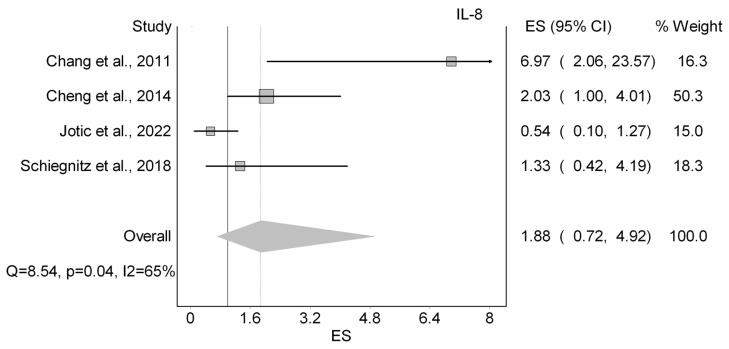
Forest plot summarizing the effect of Serum IL-8 on overall survival [44,52,53,65].

Eight papers (20%) explored the prognostic parameters of serum interleukin 8 (IL-8) in HNCs, as shown in Table 2. Five studies evaluated OS, and two evaluated DFS. Regarding OS, four out of five studies reported HRs were not statistically significant (Figure 2) [44,53,63,65]. It should be noted that Jotic et al. (2022) was the only study to report a better prognosis associated with higher expression of IL-8, with an HR of 0.54 [65]. Our analysis showed a nonsignificant worsting in survival with a HR of 1.88 (95%CI 0.72–4.92; I^2^: 65%; LFK: −0.69;. Moreover, discrepancies were observed for DFS. While Cheng et al. (2014) demonstrated a significant HR for DFS of 1.94 (95% CI: 1.03–3.32) [53], Fujita et al. (2014) reported the HR of DFS as nonsignificant being 1.07 (95% CI: 0.54–2.13) [35]. Jotic et al. (2022) also investigated DSS and reported a statistically significant HR of 0.18 (95% CI: 0.35–0.94) [65]. Furthermore, CSS and RFS were examined by two different studies. They both showed statistically significant HRs of 1.2 (95% CI: 1.1–1.3) and 7.45 (95% CI: 2.40–23.15) [59,60], respectively. Lastly, Hao et al. (2013) reported a nonsignificant HR of 1.35 for PFS [63]. All in all, serum IL-8 showed insignificant prognostic value, with conflicting associations across outcomes.

#### 3.3.2. CXCL10

**Figure 3:**
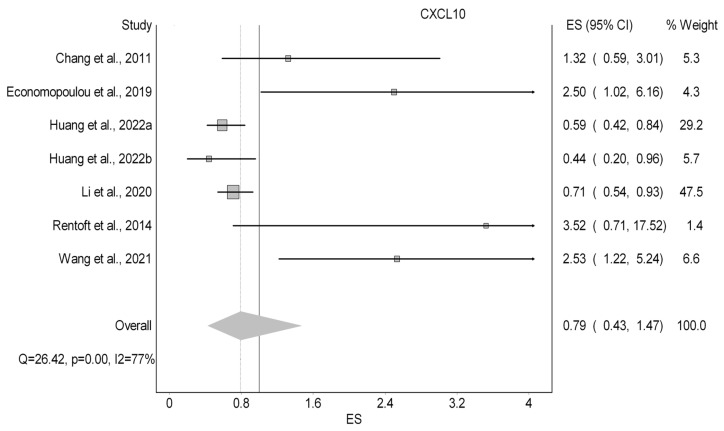
Forest plot summarizing the effect of CXCL10 on overall survival [39,52,58,66,67,72].

The prognostic parameters of CXCL10 were investigated in six studies (15%) as displayed in Table 3. The studies used two different types of samples, with tissue samples being the most common. The OS was evaluated by all six papers. However, PFS and DFS were each studied once in different papers. As for the OS, our quantitative analysis (Figure 3) showed an nonsignificant improvement in OS with a HR of 0.79 (95%CI 0.43–1.47; I^2^: 77%; LFK: 4.47). Economopoulou et al. (2019) found a significant HR of PFS of 2.42 (95% CI: 1.08–5.45) [66]. In another study, Li et al. (2020) noted the HR of DFS to be 2.42 and non-significant (95% CI: 0.84–6.99) [67]. 

#### 3.3.3. CXCR4

**Figure 4:**
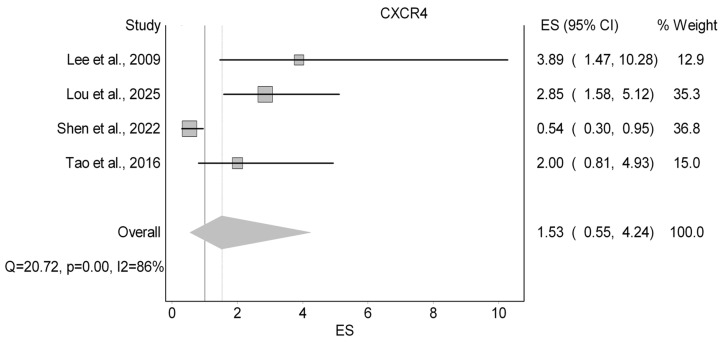
Forest plot summarizing the effect of CXCR4 on overall survival [40,57,69,71].

The six studies (13.6%) that examined CXCR4 from tissue samples as a prognostic marker were depicted in Table 4. Among them, four papers investigated OS (Figure 4), two investigated LRC, and one investigated DFS. Starting with OS, our analysis revealed an insignificant increase in the HR of OS with a HR of 1.53 (95%CI 0.55–4.24; I^2^: 86%; LFK: −38). Next, both papers examining LRC of cytoplasmic CXCR4 showed non-significant HRs of 1.13 (95% CI: 0.52–2.46) [61] and 3.81 (95% CI: 0.92–15.78) [62]. Finally, Tao et al. (2016) studied DFS and observed a nonsignificant HR of 2.26 (95% CI: 0.83–6.20) [57].

#### 3.3.4. MIP-3α

Three studies explored MIP-3 α (macrophage inflammatory protein-3 alpha) as a prognostic marker as shown in Table 5. Chang et al. (2008) and Chang et al. (2011) studied the OS of serum MIP-3 α showing a statistically significant HR of 4.27 (95% CI: 2.44–7.49), and 7.38 (95% CI: 2.19–24.86), respectively [51,52]. Furthermore, Chang et al. (2008) examined MFS, showing a statistically significant HR of 2.54 (95% CI: 1.41–4.58) [51]. Finally, DMFS, using tissue MIP-3α, was assessed by Tang et al. (2015), which reported a significant HR of 8.10 (95% CI: 1.82–35.93) [56]. Collectively, MIP-3α consistently demonstrates a significant correlation with poor survival outcomes, suggesting its strong prognostic potential.

### 3.4. Diagnostic Performance of Chemokines

Table S4 shows the diagnostic performance of CXCL10, eotaxin, MIP-1B, HCC-1, PF-4, and CCL-27. Although the diagnostic performance was assessed for various chemokines in the literature, the most frequently investigated chemokines were IL-8 and chemerin.

#### 3.4.1. Salivary and Serum IL-8

Table 6 illustrates the sensitivity and specificity of serum and salivary IL-8 among participants with OSCC, highlighting its diagnostic potential. Among the 14 studies assessing the diagnostic performance of chemokines, eight provided data on salivary IL-8 [30,37,38,41,43,45,46,74] while three focused on serum IL-8 [43,44]. Disparities in the sensitivity and specificity were noted across the studies. The highest specificity (100.00%) was recorded by both Singh et al. (2020) using salivary IL-8 and Hamad et al. (2011) using serum IL-8 [38,45]. Conversely, the lowest specificity of 56.67% was documented by Gleber-Netto et al. (2016) using salivary IL-8 [37]. As for sensitivity, the highest value of 98.28% was achieved by Singh et al. (2020) based on salivary IL-8 [45], while the lowest value of 58.00% was reported by Rajkumar et al. (2014) based on serum IL-8 [43].

**Figure 5:**
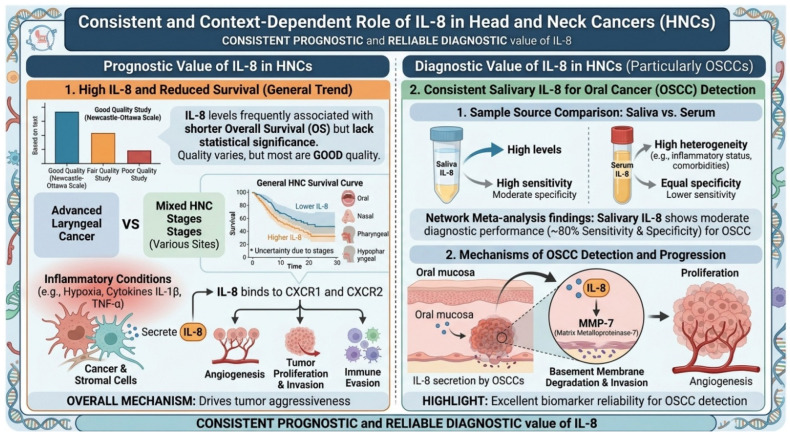
Prognostic and diagnostic roles of interleukin-8 (IL-8) in head and neck cancers (HNCs). This schematic summarizes the clinical relevance of IL-8 in HNCs. Elevated IL-8 levels, measured in saliva or serum, are associated with poorer overall survival, advanced tumor stage, and inflammatory tumor microenvironment. IL-8 signals through CXCR1 and CXCR2 to promote angiogenesis, tumor cell proliferation, invasion, and immune modulation. Diagnostic utility is highlighted particularly in oral squamous cell carcinoma (OSCC), where salivary IL-8 demonstrates high sensitivity and moderate specificity compared with serum. Mechanistically, IL-8 contributes to OSCC progression via matrix metalloproteinase (MMP) activation, basement membrane degradation, and enhanced angiogenesis. Overall, IL-8 represents a consistent and reliable biomarker with both prognostic and diagnostic value in HNCs. A preliminary visualization was generated using GPIA to represent the initial data; it was subsequently extensively revised by the corresponding author to ensure accuracy and appropriate formatting.

A comparison between salivary and serum IL-8 by Rajkumar et al. (2014) revealed that salivary IL-8 exhibited a markedly superior ability to distinguish OSCC cases from controls. Specifically, salivary IL-8 achieved a sensitivity of 85.00% and a specificity of 93.00%, highlighting its robustness as a diagnostic biomarker. In contrast, serum IL-8 demonstrated lower values, with a sensitivity of only 58.00% and a specificity of 72.00%, suggesting that salivary IL-8 may provide a more reliable and practical option for clinical use in diagnosing OSCC [43]. Salivary and serum IL-8 displayed variable diagnostic performance, with salivary IL-8 generally showing a higher sensitivity and specificity. This highlights IL-8 as a reliable diagnostic marker particularly in salivary samples (Figure 5).

#### 3.4.2. Salivary and Serum Chemerin

Salivary chemerin’s diagnostic performance in individuals with OSCC was evaluated in two studies [36,47]. The findings of these studies were consistent, as both reported a sensitivity of 100% and a specificity of 100% for salivary chemerin as a diagnostic biomarker [36,47]. Both Ghallab and Shaker (2017) [36] as well as Susha and Ravindran (2023) [47] employed closely aligned cutoff values of >6.92 ng/mL and 6.55 ng/mL, respectively. Additionally, Ghallab and Shaker (2017) extended their analysis to assess the performance of serum chemerin as a diagnostic biomarker. They reported identical sensitivity and specificity rates of 100%, comparable to salivary chemerin, albeit using a majorly higher cutoff value of 307 ng/mL for serum chemerin. These findings collectively underscore the potential of chemerin, both in saliva and serum, as a robust biomarker for non-invasive detection of OSCC. However, it is important to note that these findings are derived from a very limited number of studies with relatively small sample sizes. Such perfect diagnostic estimates (100% sensitivity and specificity) are uncommon in clinical research and may reflect potential overestimation due to study design limitations, selection bias, or lack of external validation. Thus, while chemerin shows promising diagnostic performance, the evidence remains limited and needs further validation.

### 3.5. Quality Assessment

Two quality assessment tools were utilized. First, the Newcastle–Ottawa Scale (NOS) was used to examine cohort studies, as illustrated in the appendix (Table S5). This tool uses a star system to evaluate three domains: selection, comparability, and outcome. Most of the included studies in this systematic review were classified as good quality. Conversely, only seven out of the 31 included cohort studies exhibited poor quality. The second assessment tool was the Quality Assessment of Diagnostic Accuracy Studies (QUADAS-2), shown in the appendix (Table S6). It is an assessment tool applied to diagnostic studies that examines four domains: patient selection, index test, reference standard, and flow and timing. The 13 diagnostic studies included in this systematic review displayed a low risk of bias and no applicability concerns. Overall, the majority of studies included showed good methodological quality, therefore supporting the reliability of our findings.

## 4. Discussion

Chemokines are crucial in regulating immune responses and tumor progression, making them potential biomarkers for both prognostic and diagnostic purposes in cancer [14]. To further investigate their role in the diagnosis and/or prognosis of HNCs, we conducted a comprehensive systematic review of 44 studies, involving 7294 participants. Our findings indicated that high levels of IL-8 were associated with lower survival rates, but the difference is not statistically significant. CXCL10 showed inconsistent results, with some studies linking it to better or worse OS, as well as increased risks for PFS and DFS. CXCR4 also displayed inconsistencies, with increased risks for OS, LRC, and DFS. Finally, MIP-3α was consistently linked to poor prognosis across the studies reviewed. Notably, chemerin and IL-8 were the most extensively studied chemokines in the diagnosis of HNCs. Chemerin demonstrated a promising diagnostic performance in two relatively small studies, achieving 100% sensitivity and specificity, while IL-8 exhibited varying sensitivity and specificity across the included studies.

The overall methodological quality of the studies included supports the credibility of our findings, though some variability was noted. Most of the prognostic studies (21 out of 31) were rated as good quality based on the Newcastle–Ottawa Scale, lending robustness to the observed associations. However, the presence of seven poor-quality and three fair-quality studies introduces some risk of bias—often due to issues such as inadequate comparability between groups, insufficient follow-up, or unclear selection methods—which should be considered when interpreting trends, especially for chemokines with inconsistent findings. In contrast, all 11 diagnostic studies assessed using the QUADAS-2 tool demonstrated a low risk of bias with no applicability concerns, which strengthens our conclusions regarding the diagnostic potential of biomarkers like chemerin and IL-8. Taken together, these quality assessments provide critical context for understanding the prognostic and diagnostic roles of chemokines in HNCs.

In our review, we observed that for each chemokine, different studies employed varying detection techniques, including ELISA, RT-qPCR, immunohistochemistry (IHC), and multiplex assays. This methodological heterogeneity may contribute to the variability in reported diagnostic and prognostic outcomes across studies. For instance, ELISA and multiplex assays detect protein levels, reflecting post-transcriptional modifications and protein abundance, while RT-qPCR measures mRNA expression, which may not directly correlate with protein levels due to regulatory mechanisms [75,76]. IHC provides spatial localization of proteins within tissues but is semi-quantitative and highly dependent on antibody specificity and tissue processing [77]. These differences in detection modalities can influence the sensitivity and specificity of chemokine measurements. Therefore, the observed discrepancies in chemokine-related outcomes across studies may, in part, be attributed to the differences in detection techniques employed, emphasizing the need for methodological standardization in future research.

Altogether, this review illustrates that chemokines exhibit variable prognostic and diagnostic roles in HNCs, with IL-8 and MIP-3α showing more consistent prognostic associations, while chemerin and IL-8 provide a potential diagnostic value, but heterogenicity across studies contributes to the variability in outcomes reported.

### 4.1. Role of Chemokines and/or Their Receptors in the Prognosis of Head and Neck Cancers

Many chemokines have been associated with cancer progression and prognosis in the literature. IL-8 has been frequently studied for its role in tumor progression and metastasis. Although most included studies showed that IL-8 was associated with reduced OS in patients with HNC, Jotic et al. (2022) identified IL-8 as a significant predictor of 3-year OS and disease-specific survival (DSS) in patients with advanced laryngeal cancer [65]. This discrepancy could be explained by the fact that Jotic et al. included only patients with advanced laryngeal cancer, whereas other studies included patients at various stages of the disease. Additionally, differences in tumor biology, treatment response, and cytokine interactions in advanced stages may influence the prognostic role of IL-8. These findings highlight the importance of understanding the multifaceted role of IL-8 in tumor progression and its complex prognostic implications. IL-8, also known as CXCL8, is a pro-inflammatory chemokine secreted by tumor and stromal cells under inflammatory conditions like hypoxia and cytokines (e.g., IL-1β, TNF-α), acting via CXCR1 and CXCR2 receptors [78]. These receptors activate pathways (PI3K/Akt, MAPK, NF-κB) promoting tumor angiogenesis, proliferation, invasion, and immune evasion [78,79]. IL-8 also facilitates extracellular matrix degradation and tumor vascularity through endothelial proliferation and MMP expression [78], leading to greater tumor aggressiveness and worse survival outcomes [80].

Recent studies have increasingly highlighted the role of CXC chemokines and their receptors in regulating the tumor microenvironment, cancer cell proliferation, and metastasis [67]. In our study, we identified inconsistencies in the OS results associated with CXCL10. For instance, Li et al. (2021) reported that increased CXCL10 expression was associated with better OS rates in patients with head and neck squamous cell carcinoma (HNSCC), suggesting a potentially favorable role in immune response and tumor suppression [67]. Conversely, Rentoft et al. (2014) found that high levels of CXCL10 were linked to poor OS in patients with squamous cell carcinoma of the tongue [72]. Similarly, our results showed that CXCR4 demonstrated contrasting findings with OS. Despite the discrepancy, a meta-analysis by Zhao et al. (2015) revealed that the overexpression of CXCR4 in patients with HNC was associated with worse OS rates [81].

A number of these conflicting findings may be elucidated by the paradoxical role of CXCL10 and CXCR4 in head and neck cancers. CXCL10, also known as interferon-inducible protein-10, exhibits a bifunctional nature, acting both as an immune recruiter and as a promoter of tumor aggressiveness. While it generally serves to recruit cytotoxic immune cells, such as T lymphocytes and natural killer (NK) cells [82], its overexpression in HNC is closely associated with more aggressive tumor behavior, immune evasion, and diminished progression-free survival [58,66]. Upon binding to its receptor (CXCR3), CXCL10 activates pivotal signaling cascades, including JAK/STAT, ERK1/2, and p38 MAPK, thereby promoting tumor cell proliferation, migration, and epithelial–mesenchymal transition (EMT) [82,83]. Similarly, CXCR4 also exhibits dual functionality; in certain malignancies, its activation may induce apoptosis of tumor cells or inhibit their proliferation, thereby functioning as a tumor suppressor [84]. However, in the context of HNCs, elevated CXCR4 expression has been shown to be correlated with advanced tumor stages and poor survival outcomes through mediating the chemotaxis of tumor cells and their metastatic dissemination to CXCL12-enriched microenvironments [81], such as lymph nodes, lungs, and liver [85,86]. These roles suggest that the prognostic significance of CXCL10 and CXCR4 may vary depending on the cancer subtype as well as the variations within the tumor microenvironment. Factors such as tumor stage and treatment modality may contribute to the observed variability in outcomes, emphasizing the need for further research to understand their context-dependent effects.

Moreover, elevated MIP-3α levels have been consistently linked, in the included studies, to poor survival rates in HNC patients, indicating its potential as a prognostic biomarker. MIP-3α, also known as CCL20, secreted by tumor cells, stromal cells, and TAMs, binds to CCR6 to modulate the tumor microenvironment [87,88]. Our findings could be explained by the role MIP-3α plays in the pathogenesis of the HNCs. First, MIP-3α recruits immunosuppressive CCR6+ cells, such as immature dendritic cells and regulatory T cells, suppressing antitumor immunity [87]. Additionally, it enhances EMT and tumor invasion, further exacerbating the survival rates in HNC patients [89].

In general, these findings indicate IL-8 and MIP-3α capability as a prognostic marker, as it was consistently correlated with poorer survival. On the other hand, CXCL20 and CXCR4 showed conflicting prognostic effects, possibly due to the influence of tumor subtype and microenvironmental factors.

### 4.2. Role of Chemokines in the Diagnosis of Head and Neck Cancers

Our results reveal notable differences in the diagnostic performance between IL-8 and Chemerin. Although both chemokines have been implicated in cancer cell proliferation and angiogenesis, underscoring their high diagnostic potential [90], chemerin remains relatively understudied compared to IL-8.

Our findings suggest that salivary IL-8 is a promising biomarker for diagnosing HNCs, particularly OSCC (Figure 5). This is supported by studies consistently demonstrating higher salivary IL-8 levels in HNC patients compared to healthy controls [91,92]. For example, a network meta-analysis of 40 studies observed that patients with oral cancer had higher salivary IL-8 levels compared to healthy controls, demonstrating a moderate diagnostic performance with 80% sensitivity and specificity [93]. The strong diagnostic potential of IL-8 stems from its multifaceted role within the tumor microenvironment, particularly in the progression of HNCs. IL-8 secretion by OSCCs has been shown to upregulate matrix metalloproteinase-7 (MMP-7), a key mediator of invasion, while also promoting proliferation, angiogenesis, and the recruitment of immunosuppressive cells [93,94,95]. These diverse mechanisms highlight IL-8’s exceptional reliability as a biomarker for detecting HNCs, especially OSCC.

Upon comparing serum and salivary IL-8, we observed that both exhibited a similar specificity; however, salivary IL-8 showed a greater sensitivity, underscoring its diagnostic advantage for HNCs. In line with our findings, Rezaei et al. (2019) found that salivary IL-8 concentrations of patients with OSCC were 4.8-fold higher than serum levels in their meta-analysis [91]. It has been suggested that serum IL-8 levels may be influenced by the heterogeneity of HNC, including factors such as tumor progression, inflammatory status, or comorbidities, which limits its reliability as a standalone diagnostic marker [96]. Hence, salivary IL-8 emerges as a more valuable and stable diagnostic tool, offering a non-invasive, easily accessible, and potentially more reflective measure of local tumor activity and inflammatory responses in the head and neck region.

The relatively high sensitivity and specificity observed for salivary IL-8 in the diagnosis of OSCC highlight its potential clinical relevance as a biomarker. From a translational perspective, IL-8 is particularly attractive due to its detectability in saliva, a biofluid that can be obtained non-invasively. This positions salivary IL-8 as a promising candidate for use in screening and early detection strategies, particularly in high-risk populations. In terms of clinical application, IL-8 may support early detection of OSCC and could be incorporated into multimarker panels to improve diagnostic accuracy when combined with other tumor-related biomarkers. However, given the variability observed across studies, IL-8 is unlikely to function effectively as a standalone biomarker and should instead be considered as part of an integrated diagnostic framework requiring further validation.

Furthermore, despite the limited number of available studies, our paper highlights chemerin as a potentially highly specific and sensitive biomarker for OSCC, although current evidence remains limited. Chemerin achieved 100% specificity and sensitivity across both salivary and serum samples. These findings align with other studies [36,97], including Khijmatgar et al. (2024), who identified chemerin as a top-performing biomarker for diagnosing OSCC with a sensitivity of 0.94 (95% CI: 0.78–1.00) [98]. Chemerin is a multifunctional adipokine with established roles in inflammation, adipogenesis, and glucose homeostasis [99]. It acts primarily through its receptors CMKLR1 and GPR1, which mediate signaling pathways, including RhoA/ROCK, involved in tumor cell proliferation, migration, invasion, and immune modulation [100,101,102]. The diagnostic relevance of chemerin is highlighted by its elevated levels in biological fluids like saliva in patients with HNCs, particularly OSCCs [103]. This elevation is associated with key tumor-related processes, such as extracellular matrix remodeling and inflammatory cytokine production [99]. Additionally, chemerin’s role in recruiting immune cells, such as macrophages and dendritic cells, further supports its utility in differentiating cancerous from non-cancerous states [100]. These characteristics highlight chemerin as a promising candidate for early detection and diagnostic evaluation in HNCs. Despite these promising findings, several important caveats warrant consideration and cautious interpretation. First, the evidence base for chemerin as a diagnostic biomarker remains limited, with only two small primary studies reporting perfect diagnostic accuracy (*n* = 30 and *n* = 64 in Ghallab and Shaker (2017) and Susha and Ravindran (2023), respectively) [36,47]. Such perfect sensitivity and specificity values are uncommon in biomarker research and may be overestimated due to small sample sizes, potential selection bias, or overfitting, particularly in the absence of validation cohorts. Second, both studies were conducted in specific populations (Egypt and India), and their findings have not yet been validated in larger, independent, multi-center cohorts, limiting generalizability. Third, the absence of external validation cohorts, along with the lack of standardized cutoff values across populations, further constrains the reliability and reproducibility of these results. Additionally, the included studies did not stratify findings by tumor stage or histological grade, leaving uncertainty regarding chemerin’s diagnostic performance across different disease stages. Therefore, while chemerin shows promise as a potential non-invasive biomarker for OSCC detection, these findings should be interpreted as preliminary, and larger prospective studies are needed to validate its diagnostic utility before clinical implementation.

Given that OSCC represented the most frequently investigated tumor subtype in this review, these mechanistic findings are particularly relevant to oral carcinogenesis. OSCC development is strongly influenced by chronic inflammation and tumor microenvironment interactions, where chemokine signaling regulates immune cell recruitment, angiogenesis, epithelial–mesenchymal transition (EMT), and extracellular matrix remodeling. IL-8 promotes OSCC progression through activation of CXCR1/2-mediated pathways that enhance tumor cell proliferation, invasion, and vascularization, while CXCL10 and CXCR4 contribute to immune modulation and metastatic dissemination through tumor–immune crosstalk. Similarly, CCL20 (MIP-3α) has been implicated in OSCC progression through recruitment of CCR6+ regulatory immune cells that facilitate immune evasion and tumor persistence. These findings highlight the biological relevance of chemokine-mediated signaling networks in OSCC pathogenesis and support their investigation as mechanistically informative biomarkers in oral cancer research [81,85].

#### 4.2.1. Context-Dependent Performance of Chemokine Biomarkers Across HNC Subtypes

Notably, the diagnostic and prognostic performance of chemokines varied not only between biomarkers but also across tumor subtypes and sample sources, underscoring the biological heterogeneity of HNC. IL-8, for instance, demonstrated relatively consistent adverse prognostic associations across multiple subtypes, including nasopharyngeal carcinoma (NPC) [53], oral squamous cell carcinoma (OSCC) [35], and laryngeal squamous cell carcinoma (LSCC) [63], suggesting a more universal role in promoting tumor progression and inflammation-driven oncogenesis.

In contrast, CXCL10 and CXCR4 exhibited more context-dependent and, at times, conflicting effects. Elevated CXCL10 expression was associated with improved overall survival in OSCC (HR 0.44–0.59) [39], yet correlated with poorer outcomes in NPC (HR 2.53) [58] and HNSCC (HR 2.5) [66]. Similarly, CXCR4 showed divergent prognostic associations, ranging from protective effects (HR 0.54) [69] to markedly increased risk (HR 2.00–3.89) [40,57]. These inconsistencies likely reflect fundamental differences in tumor biology, including etiological drivers such as human papillomavirus (HPV) in oropharyngeal cancers and Epstein–Barr virus (EBV) in NPC, which differentially shape immune infiltration, chemokine signaling, and tumor–host interactions.

Beyond tumor subtype, sample source also appears to significantly influence biomarker performance. Tissue-based measurements, particularly for CXCL10, were more consistently associated with survival outcomes compared to circulating levels [39,58,67,72], potentially due to their closer reflection of the localized tumor microenvironment, where chemokine-mediated signaling is most active. This highlights an important methodological consideration, as variability in biospecimen type may contribute to heterogeneity in reported findings across studies.

In contrast to these more extensively studied chemokines, chemerin has been investigated exclusively in OSCC, limiting its applicability to other HNC subtypes and further emphasizing the need for broader evaluation across diverse tumor contexts.

Collectively, these observations reinforce that chemokine biomarkers cannot be interpreted in isolation. Rather, their clinical relevance is highly context-dependent, influenced by tumor subtype, anatomical sites, etiological factors, and methodological differences such as sample source. Future research should therefore prioritize standardized, subtype-stratified analyses and integrative approaches that account for tumor biology, in order to more accurately define the diagnostic and prognostic utility of chemokines in HNC.

#### 4.2.2. Heterogeneity Across Studies and Methodological Considerations

A major finding of this review is the substantial heterogeneity across included studies, complicating direct comparison of results. This heterogeneity was evident across multiple dimensions, including tumor subtypes (OSCC, NPC, LSCC, OPSCC, HNSCC), which differ in molecular profiles and etiologies; sample sources (tissue, serum, saliva, plasma), where biomarker concentrations vary between local and systemic compartments; and outcome measures (OS, DFS, PFS, CSS, DSS, LRC), with differing definitions and follow-up durations. A particularly important contributor to this variability is the wide range of analytical platforms employed, including ELISA, multiplex suspension arrays, immunohistochemistry (IHC), RT-qPCR, gene expression profiling, and immunofluorescence. These techniques differ fundamentally in what they measure. For example, protein-based assays (ELISA, multiplex) quantify circulating or secreted chemokines in biofluids, while RNA-based approaches (RT-qPCR, RNA-seq) measure mRNA transcript abundance, which may not correlate with protein levels due to post-transcriptional regulation, and IHC provides spatial localization but remains semi-quantitative and subject to interpretation variability [75,76,77].

These methodological differences have direct implications for biomarker performance. For instance, tissue-based assessments of CXCL10 using mRNA or IHC demonstrated more consistent prognostic associations, whereas serum-based measurements showed more variable outcomes [39,52,58,66,67,72]. Similarly, IL-8 diagnostic studies employed both ELISA and multiplex assays in saliva, contributing to the wide range of reported sensitivity (58–98%) and specificity (57–100%) [33,37,38,41,43,45,46].

The observed inconsistencies in prognostic associations for IL-8, CXCL10, and CXCR4 may also reflect the complex and dynamic nature of the tumor microenvironment in head and neck cancers. Chemokine expression is influenced not only by tumor cells but also by stromal and immune components, including macrophages, neutrophils, and endothelial cells, particularly under inflammatory or hypoxic conditions [78,79]. For example, IL-8 is produced by multiple cell types within the tumor microenvironment and may reflect both tumor aggressiveness and host inflammatory responsesv [78,79,80]. Similarly, CXCL10 may exhibit context-dependent effects through recruitment of cytotoxic immune cells while also contributing to chronic inflammation and immune dysregulation, potentially explaining its dual prognostic associations [82,83]. CXCR4 signaling is likewise influenced by ligand gradients and microenvironmental interactions that regulate tumor cell migration and metastatic dissemination [85,86]. In addition, systemic inflammatory conditions and comorbidities may affect circulating chemokine levels, particularly in serum-based studies, further contributing to variability across findings [91,96]. Collectively, these observations suggest that heterogeneity in reported associations reflects both biological complexity and methodological differences, which limits comparability across studies, precludes meaningful quantitative synthesis, and complicates the identification of clinically reliable biomarkers. Future research should prioritize methodological harmonization, including standardized sample collection protocols and validated assays, to enable meta-analytic synthesis and clinical translation, while accounting for context-specific tumor microenvironment and immune-related influences on chemokine expression in HNCs.

### 4.3. Implications and Future Research

The findings from this systematic review highlight the complex and multifaceted roles of chemokines and their receptors in the diagnosis and prognosis of HNCs. By identifying key biomarkers, such as IL-8, CXCL10, CXCR4, MIP-3α, and chemerin, our findings pave the way for future studies to focus on these potentially useful chemokines in HNCs.

From a translational perspective, OSCC represents a particularly suitable model for chemokine biomarker development due to the accessibility of oral lesions and the feasibility of repeated saliva-based sampling. Salivary biomarkers are especially attractive in OSCC because saliva is in direct contact with tumor tissue, allowing detection of locally secreted inflammatory mediators and tumor-derived molecules [104]. This proximity may partly explain the relatively strong diagnostic performance observed for salivary IL-8 and chemerin in OSCC populations. The integration of salivary chemokine biomarkers into clinical practice could potentially support earlier detection of oral malignancies, improve risk stratification of premalignant lesions, and enable non-invasive monitoring of disease progression or treatment response. Therefore, further validation of chemokine panels in OSCC-specific cohorts may facilitate the development of clinically applicable biomarker strategies in oral oncology therapeutically, targeting chemokine signaling pathways represents a promising strategy for mitigating tumor progression and metastasis. For example, the U.S. Food and Drug Administration has approved AMD3100 (plerixafor, Mozobil^®^), a CXCR4 antagonist, while several additional agents targeting chemokine pathways are currently under investigation in clinical trials [81,105]. Additionally, combining chemokine-targeted therapies with immunotherapy could potentially enhance anti-tumor immune responses by modulating the tumor microenvironment, creating synergistic therapeutic effects [106]. This is validated by a recent study conducted by Yoshida et al. (2024) [107] ho investigated the effects of adding the CXCR4 inhibitor AMD3100 to cisplatin in OSCC cells in vitro and in mouse xenograft models in vivo. The study revealed that this combination improved the anti-tumor effect of cisplatin and reduced the number of CXCR4-positive blood vessels in cisplatin-resistant OSCC xenografts. These findings suggest that the addition of a CXCR4 inhibitor may enhance the anti-tumor effects of cisplatin in patients with refractory OSCC [107].

Conducting longitudinal studies will be critical for understanding the dynamic changes in chemokine expression during disease progression and treatment, enabling the development of personalized therapeutic strategies. Future research should prioritize large-scale prospective studies to validate the diagnostic utility of chemokines at different stages of HNC. The therapeutic potential of targeting chemokine pathways, such as the CXCL12-CXCR4 axis, also warrants further investigation. Furthermore, meta-analyses to validate the prognostic significance of chemokines across diverse populations and clinical settings will further solidify their role in improving the management and treatment of HNCs.

All in all, these findings underline the clinical potential of chemokines as diagnostic and prognostic biomarkers in HNCs and emphasize the need for future research to support and validate their utility in large-scale studies.

### 4.4. Limitations

There are several limitations that must be acknowledged. First, the review is confined to studies published in the English language, potentially excluding relevant research published in other languages, which could introduce language bias. Second, although a meta-analysis was performed, it was limited to a small subset of chemokines and restricted to OS outcomes. The included studies demonstrated substantial heterogeneity, as reflected by the high I^2^ values observed in our analyses. Potential sources of this heterogeneity include differences in tumor subtypes (e.g., OSCC, NPC, OPSCC), sample types (saliva, serum, and tissue), study designs, patient populations, tumor stages, and methodological variations, including differences in analytical and technical techniques used among the included studies. These factors likely contributed to the observed variability and limit the interpretability of the pooled results. Furthermore, the small number of studies contributed to wide confidence intervals and statistically non-significant findings, raising the possibility of type II error. There is also a potential risk of publication bias, although this could not be reliably assessed due to the limited number of studies included in each synthesis. Subgroup and sensitivity analyses were not performed because of the small number of studies and limited variability in reported variables. Another important limitation is the clinical heterogeneity of tumor types included in this review, encompassing oral cavity, oropharyngeal, nasopharyngeal, and laryngeal cancers. These entities differ in their underlying biology, including molecular drivers, HPV status, and tumor immune microenvironment. Due to the limited number of studies available for each chemokine within individual tumor subtypes, stratified or subgroup analyses by tumor site were not feasible. This may limit the interpretability and generalizability of the findings, and future research should aim to provide more standardized, site-specific data to allow more precise analyses. Furthermore, a meta-analysis of diagnostic performance was not feasible, as most studies reported only sensitivity and specificity without sufficient data to reconstruct 2 × 2 contingency tables. Additionally, the included studies themselves have inherent limitations, as many of the studies included in this review are case–control or retrospective cohort studies, which are inherently at risk of selection bias. Such study designs, combined with small sample sizes and varying quality, further constrain the generalizability of the results. Moreover, it is important to note that most of the studies were conducted in China, which may restrict the external validity of the findings to other populations. Additionally, the lack of stratification by Human Papillomavirus (HPV) status, especially in oropharyngeal cancer, is a key limitation, as HPV-positive tumors have distinct chemokine profiles and prognosis. Lastly, due to inconsistent tumor site reporting, we could not perform site-specific analyses; nasopharyngeal carcinoma was included for completeness but differs significantly from other HNC subtypes. Future studies should stratify by HPV status and tumor site to improve biomarker specificity and clinical relevance.

## 5. Conclusions

This systematic review and meta-analysis provide comprehensive evidence that chemokines play important but context-dependent roles in the diagnosis and prognosis of HNCs. MIP-3α demonstrated the most consistent association with poorer survival outcomes, identifying it as a particularly promising prognostic biomarker. In contrast, pooled analyses for IL-8, CXCL10, and CXCR4 showed heterogeneous and predominantly non-significant effects, reflecting the complex and subtype-specific nature of chemokine signaling within the tumor microenvironment. From a diagnostic perspective, salivary IL-8 demonstrated relatively consistent accuracy for oral squamous cell carcinoma, while chemerin showed very high diagnostic performance in preliminary studies. Despite promising signals, substantial heterogeneity across tumor subtypes, analytical platforms, and biospecimen sources highlights the need for standardized, large-scale prospective validation. Future integrative research combining biomarker validation with therapeutic targeting of chemokine pathways may advance precision oncology approaches in HNCs.

## Figures and Tables

**Table 1:** Characteristics of included studies.

First Author	Country	Sample Size (*n*)	Male (%)	Median/Mean Age	Tumor Type	Technique	Sample Type
OSCC							
Adamov et al., 2024 [30]	Bulgaria	107	58.9	54.2	OSCC	Fluorescent MBA	Saliva
Chen et al., 2024 [31]	China	671	69	<70: 50%	OSCC	RT-qPCR	Tissue
Chang et al., 2013 [32]	Taiwan	211	88.6	49.2	OSCC	ELISA	Serum
Dikova et al., 2021 [33]	Spain	157	50	-	OSCC	MBA	Saliva
Domingueti et al., 2019 [34]	Chile, Brazil	124	66.1	62	OSCC	IHC	Tissue
Fujita et al., 2014 [35]	Japan	50	64.1	68.6	OSCC	ELISA	Serum
Ghallab and Shaker, 2017 [36]	Egypt	30	36.6	47.6	OSCC	ELISA	Serum
Gleber-Netto et al., 2016 [37]	Taiwan	180	93.8	50.9	OSCC	ELISA	Saliva
Hamad et al., 2011 [38]	Iraq	50	53.3	22–84	OSCC	ELISA	Saliva
Huang et al., 2022 [39]	USA	342	69	61	OSCC	Gene Set Enrichment Analysis	Tissue
Lee et al., 2009 [40]	South Korea	74	75.6	59.2	OSCC	IHC	Tissue
Lee et al., 2018 [41]	Taiwan	65	86.2	29 (>55)	OSCC	Bead-based Multiplex Assay	Plasma
Mao et al., 2020 [42]	China	102	52.9	(≥60) 50%	OSCC	IHC	Tissue
Rajkumar et al., 2014 [43]	India	200	66.5	-	OSCC	ELISA	Serum
Schiegnitz et al., 2018 [44]	Germany	130	51.5	-	OSCC	ELISA	Serum
Singh et al., 2020 [45]	India	129		-	OSCC	ELISA	Saliva
St John et al., 2004 [46]	USA	64		-	OSCC	qRT-PCR/ELISA	Saliva
Susha and Ravindran, 2023 [47]	India	64	81.3	-	OSCC	ELISA	Saliva
Tsuzuki et al., 2006 [48]	Japan	90	53	62.7	OSCC/OPSCC	IHC	Tissue
Yanagiya et al., 2021 [49]	Japan	59	59.3	66	OSCC	IHC	Tissue
NPC							
Long et al., 2025 [50]	China	268	67	50.91	NPC	ELISA	Serum
Chang et al., 2008 [51]	Taiwan	166	63.8	48	NPC	ELISA	Serum
Chang et al., 2011 [52]	Taiwan	132	71.2	48.2	NPC	Multiplex suspension array system	Serum
Cheng et al., 2014 [53]	China	99	61.6	53	NPC	ELISA	Serum
Lu et al., 2011 [54]	China	297	76.4	33% (>50)	NPC	ELISA	Serum
Mao et al., 2018 [55]	China	216	66.2	50% (>45)	NPC	ELISA	Plasma
Tang et al., 2015 [56]	China	114	71.9	48	NPC	IHC	Tissue
Tao et al., 2016 [57]	China	98	60.2	46	NPC	RT-PCR	Tissue
Wang et al., 2021 [58]	China	61	52.4	50	NPC	RT-PCR	Tissue
Zergoun, 2022 [59]	Algeria	56	73.2	45	NPC	IHC	Serum
OPSCC							
Allen et al., 2007 [60]	USA	30	73.3	56	OPSCC	Immunoassay	Serum
De-Colle et al., 2017 [61]	Germany	201	80.1	57	OPSCC	IF	Tissue
De-Colle et al., 2018 [62]	Germany	141	84.4	58	OPSCC	IF	Tissue
LSCC							
Hao et al., 2013 [63]	China	92	93.5	<60: 60.9%	LSCC	ELISA	Serum
Sun et al., 2011 [64]	China	65	98.5	60	LSCC	IHC	Tissue
Jotic et al., 2022 [65]	Serbia	52	90.4	59	LC	ELISA	Serum
HNSCC							
Economopoulou et al., 2019 [66]	Greece	73	75.3	64.1	HNSCC	ELISA	Serum
Li et al., 2020 [67]	China	499		-	HNSCC	mRNA	Tissue
Li et al., 2022 [68]	China	124	62.9	-	HNSCC	RT-qPCR	Tissue
Shen et al., 2022 [69]	Germany/USA	894	75.3	60	HNSCC	HTSeq FPKM	Tissue
Wang et al., 2024 [70]	China	483	73	60	HNSCC	H&E	Tissue
TSCC							
Lou et al., 2025 [71]	China	87	59.80	60	TSCC	IHC	Tissue
Rentoft et al., 2014 [72]	Sweden	38	55	50	TSCC	qPCR	Tissue
Wang et al., 2019 [73]	China	109	67.9	54	TSCC	IHC	Tissue

Abbreviations: ELISA, enzyme-linked immunosorbent assay; GSEA, gene set enrichment analysis; H&E, hematoxylin and eosin staining; HNSCC, head and neck squamous cell carcinoma; HTSeq FPKM, high-throughput sequencing fragments per kilobase of transcript per million mapped reads; IF, immunofluorescence; IHC, immunohistochemistry; LC, laryngeal carcinoma; LSCC, laryngeal squamous cell carcinoma; MBA, multiplex bead assay; mRNA, messenger ribonucleic acid; NPC, nasopharyngeal carcinoma; OPSCC, oropharyngeal squamous cell carcinoma; OSCC, oral squamous cell carcinoma; qPCR, quantitative polymerase chain reaction; RT-qPCR, reverse transcription quantitative polymerase chain reaction; SCC, squamous cell carcinoma; TSCC, tongue squamous cell carcinoma; USA, United States of America.

**Table 2:** Serum IL-8 biomarker prognostic findings in included studies.

Author	Tumor Type	Technique	Sample Type	HR (95% CI)
Allen et al., 2007 [60]	OPSCC	Immunoassay	Serum	CSS: 1.2 (1.1–1.3)
Chang et al., 2011 [52]	NPC	SAT	Serum	OS: 6.97 (2.06–23.57)
Cheng et al., 2014 [53]	NPC	ELISA	Serum	OS: 2.03 (1.00–4.01)
				DFS: 1.94 (1.03–3.32)
Fujita et al., 2014 [35]	OSCC	ELISA	Serum	DFS: 1.07 (0.54–2.13)
Hao et al., 2013 [63]	LSCC	ELISA	Serum	OS: 1.48 *
				PFS: 1.35 *
Jotic et al., 2022 [65]	LC	ELISA	Serum	OS: 0.54 (0.10–1.27)
				DSS: 0.18 (0.35–0.94)
Schiegnitz et al., 2018 [44]	OSCC	ELISA	Serum	OS: 1.33 (0.42–4.19)
Zergoun et al., 2022 [59]	NPC	Immunoassay	Serum	RFS: 7.45 (2.40–23.15)

Abbreviations: CSS, cancer specific survival; DFS, disease-free survival; DSS, disease-specific survival; ELISA, enzyme-linked immunosorbent assay; HR, hazard ratio; LC, laryngeal carcinoma; LSCC, laryngeal squamous cell carcinoma; NPC, nasopharyngeal carcinoma; OPSCC, oropharyngeal squamous cell carcinoma; OS, overall survival; OSCC, oral squamous cell carcinoma; PFS, progression-free survival; RFS, recurrence-free survival; SAT, multiplex suspension array system. * Hazard ratios were extracted from graphs reported in studies, but no confidence intervals could be obtained.

**Table 3:** CXCL10 biomarker prognostic findings in included studies.

Author	Tumor Type	Technique	Sample Type	HR (95%CI)
Chang et al., 2011 [52]	NPC	SAT	Serum	OS: 1.32 (0.59–3.01)
Economopoulou et al., 2019 [66]	HNSCC	ELISA	Serum	OS: 2.5 (1.02–6.16)
				PFS: 2.42 (1.08–5.45)
Huang et al., 2022 * [39]	OSCC	Gene expression	Tissue	OS: 0.59 (0.42–0.84)
Huang et al., 2022 * [39]	OSCC	Gene expression	Tissue	OS: 0.44 (0.2–0.96)
Li et al., 2020 [67]	HNSCC	mRNA	Tissue	OS: 0.71 (0.54–0.93)
				DFS: 2.42 (0.84–6.99)
Rentoft et al., 2014 [72]	TSCC	qPCR	Tissue	OS: 3.52 (0.71–17.52)
Wang et al., 2021 [58]	NPC	RT-qPCR	Tissue	OS: 2.53 (1.22–5.24)

Abbreviations: DFS, disease-free survival; ELISA, enzyme-linked immunosorbent assay; HNSCC, head and neck squamous cell carcinoma; HR, hazard ratio; mRNA, messenger ribonucleic acid; NPC, nasopharyngeal carcinoma; OSCC, oral squamous cell carcinoma; OS, overall survival; PFS, progression-free survival; RT-qPCR, quantitative real-time polymerase chain reaction; SAT, multiplex suspension array system; TSCC, tongue squamous cell carcinoma. * Huang et al., 2022 used two different datasets and investigated the biomarkers in the same paper [39]. ^a^OS generated using The Cancer Genome Atlas (TCGA) oral cancer data. ^b^OS generated using GSE65858 data from Germany.

**Table 4:** CXCR4 biomarker prognostic findings in the included studies.

Author	Tumor Type	Technique	Sample Type	HR (95%CI)
De-Colle et al., 2017 [61]	OPSCC	IF	Tissue	LRC: 1.13 (0.52–2.46)
De-Colle et al., 2018 [62]	OPSCC	IF	Tissue	LRC: 3.81 (0.92–15.78)
Lee et al., 2009 [40]	OSCC	IHC	Tissue	OS: 3.89 (1.47–10.28)
Lou et al., 2025 [71]	TSCC	IHC	Tissue	OS: 2.85 (1.58–5.12)
Shen et al., 2022 [69]	HNSCC	HTSeq FPKM	Tissue	OS: 0.54 (0.30–0.95)
Tao et al., 2016 [57]	NPC	RT-PCR	Tissue	OS: 2.00 (0.81–4.93)
				DFS: 2.26 (0.83–6.20)

Abbreviations: DFS, disease-free survival; HNSCC, head and neck squamous cell carcinoma; HR, hazard ratio; HTSeq FPKM, high-throughput sequencing fragments per kilobase of transcript per million mapped reads; IF, immunofluorescence; IHC, immunohistochemistry; LRC, locoregional control; NPC, nasopharyngeal carcinoma; OPSCC, oropharyngeal squamous cell carcinoma; OS, overall survival; OSCC, oral squamous cell carcinoma; RT-PCR, quantitative real-time polymerase chain reaction.

**Table 5:** MIP-3α biomarker prognostic findings in included studies.

**Author**	**Tumor Type**	**Technique**	**Sample Type**	**HR (95%CI)**
Chang et al., 2008 [51]	NPC	ELISA	Serum	OS: 4.27 (2.44–7.49)
				MFS:2.54 (1.41–4.58)
Chang et al., 2011 [52]	NPC	SAT	Serum	OS: 7.38 (2.19–24.86)
Tang et al., 2015 [56]	NPC	IHC	Tissue	DMFS: 8.10 (1.82–35.93)

Abbreviations: HR, hazard ratio; DMFS, distant metastasis-free survival; ELISA, enzyme-linked immunosorbent assay; IHC, immunohistochemistry; MFS, metastasis-free survival; NPC, nasopharyngeal carcinoma; OS, overall survival; SAT, multiplex suspension array system.

**Table 6:** The diagnostic performance of salivary and serum IL-8 in oral squamous cell carcinoma (OSCC).

**Author**	**Technique**	**Sample Type**	**Sensitivity**	**Specificity**
Adamov et al., 2024 [30]	MBA	Saliva	70.00%	86.00%
Lee et al., 2018 [41]	MBA	Saliva	65.85%	79.17%
Dikova et al., 2021 [33]	MBA	Saliva	75.76%	92.00%
Gleber-Netto et al., 2016 [37]	ELISA	Saliva	90.00%	56.67%
Hamad et al., 2011 [38]	ELISA	Saliva	70.00%	85.00%
Rajkumar et al.,2014 [43]	ELISA	Saliva	85.00%	93.00%
Singh et al., 2020 [45]	ELISA	Saliva	98.28%	100.00%
St John et al., 2004 [46]	RT-qPCR/ELISA	Saliva	86.00%	97.00%
Hamad et al., 2011 [38]	ELISA	Serum	66.70%	100.00%
Rajkumar et al., 2014 [43]	ELISA	Serum	58.00%	72.00%
Schiegnitz et al., 2018 [44]	ELISA	Serum	74.00%	75.50%

Abbreviations: ELISA, enzyme-linked immunosorbent assay; MBA, multiplex-based assay; RT-qPCR, quantitative real-time polymerase chain reaction.

## Data Availability

The original contributions presented in this study are included in the article/Supplementary Material. Further inquiries can be directed to the corresponding author.

## Supplementary Materials

The following supporting information can be downloaded at: https://www.mdpi.com/article/10.3390/cancers18091437/s1, Table S1: Prisma checklist; Table S2: Search strategy; Table S3: Prognostic parameters of the remaining biomarkers reported in the included studies; Table S4: The diagnostic performance of the remaining chemokines; Table S5: Newcastle–Ottawa Scale evaluation of included prognostic studies; Table S6: QUADAS evaluation of included diagnostic studies.

## Abbreviations

The following abbreviations are used in this manuscript:

HNCs	Head and neck cancers
HPV	Human papillomavirus
EMT	Epithelial-to-mesenchymal transition
TP	True positives
TN	True negatives
FP	False positives
FN	False negatives
HR	Hazard ratio
OS	Overall survival
DFS	Disease-free survival
DMFS	Distant metastasis-free survival
DSS	Disease-specific survival
MFS	Metastasis-free survival
CSS	Cancer-specific survival
PFS	Progression-free survival
RFS	Recurrence-free survival
LRC	Loco-regional control
ELISA	Enzyme-linked immunosorbent assay
HNSCC	Head and neck squamous cell carcinoma
HTSeq FPKM	High-throughput sequencing fragments per kilobase of transcript per million mapped reads
IF	Immunofluorescence
IHC	Immunohistochemistry
LC	Laryngeal carcinoma
LSCC	Laryngeal squamous cell carcinoma
MBA	Multiplex-based assay
mRNA	Messenger ribonucleic acid
NPC	Nasopharyngeal carcinoma
OPSCC	Oropharyngeal squamous cell carcinoma
OSCC	Oral squamous cell carcinoma
RT-qPCR	Quantitative real-time polymerase chain reaction
SCC	Squamous cell carcinoma
USA	United States of America
IL-8	Interleukin 8
SAT	Multiplex suspension array system
RT-PCR	Quantitative real-time polymerase chain reaction
MIP-3α	Macrophage inflammatory protein-3 alpha
NOS	Newcastle-Ottawa Scale
QUADAS-2	Quality Assessment of Diagnostic Accuracy Studies
NK cells	Natural killer cells
MMP-7	Matrix metalloproteinase-7
