# Supplementary material for: Diagnostic and Prognostic Significance of Chemokines in Head and Neck Cancers: A Systematic Review and Meta-Analysis
Source: Cancers (Basel). 2026 Apr 30;18(9):1437. doi: 10.3390/cancers18091437 (PMC13162777; doi:10.3390/cancers18091437)
Supplement: Supplementary file 1 [file cancers-18-01437-s001.zip › cancers-4184413-supplementary.pdf]

Diagnostic and Prognostic Significance of Chemokines in Head and Neck Cancers: A Systematic Review and Meta-Analysis

Supplementary

Table S1: Prisma checklist

Section and Topic	Item #	Checklist item	Location where item is reported
**TITLE**			
Title	1	Identify the report as a systematic review.	1
**ABSTRACT**			
Abstract	2	See the PRISMA 2020 for Abstracts checklist.	2
**INTRODUCTION**			
Rationale	3	Describe the rationale for the review in the context of existing knowledge.	5
Objectives	4	Provide an explicit statement of the objective(s) or question(s) the review addresses.	5
**METHODS**			
Eligibility criteria	5	Specify the inclusion and exclusion criteria for the review and how studies were grouped for the syntheses.	6
Information sources	6	Specify all databases, registers, websites, organisations, reference lists and other sources searched or consulted to identify studies. Specify the date when each source was last searched or consulted.	5
Search strategy	7	Present the full search strategies for all databases, registers and websites, including any filters and limits used.	Appendix
Selection process	8	Specify the methods used to decide whether a study met the inclusion criteria of the review, including how many reviewers screened each record and each report retrieved, whether they worked independently, and if applicable, details of automation tools used in the process.	5
Data collection process	9	Specify the methods used to collect data from reports, including how many reviewers collected data from each report, whether they worked independently, any processes for obtaining or confirming data from study investigators, and if applicable, details of automation tools used in the process.	6
Data items	10a	List and define all outcomes for which data were sought. Specify whether all results that were compatible with each outcome domain in each study were sought (e.g. for all measures, time points, analyses), and if not, the methods used to decide which results to collect.	6
	10b	List and define all other variables for which data were sought (e.g. participant and intervention characteristics, funding sources). Describe any assumptions made about any missing or unclear information.	6
Study risk of bias assessment	11	Specify the methods used to assess risk of bias in the included studies, including details of the tool(s) used, how many reviewers assessed each study and whether they worked independently, and if applicable, details of automation tools used in the process.	7
Effect measures	12	Specify for each outcome the effect measure(s) (e.g. risk ratio, mean difference) used in the synthesis or presentation of results.	N/A
Reporting bias assessment	14	Describe any methods used to assess risk of bias due to missing results in a synthesis (arising from reporting biases).	N/A

Certainty assessment	15	Describe any methods used to assess certainty (or confidence) in the body of evidence for an outcome.	N/A
**RESULTS**			
Study selection	16a	Describe the results of the search and selection process, from the number of records identified in the search to the number of studies included in the review, ideally using a flow diagram.	8
	16b	Cite studies that might appear to meet the inclusion criteria, but which were excluded, and explain why they were excluded.	8, Figure 1
Synthesis methods	13a	Describe the processes used to decide which studies were eligible for each synthesis (e.g. tabulating the study intervention characteristics and comparing against the planned groups for each synthesis (item #5)).	5
	13b	Describe any methods required to prepare the data for presentation or synthesis, such as handling of missing summary statistics, or data conversions.	7
	13c	Describe any methods used to tabulate or visually display results of individual studies and syntheses.	N/A
	13d	Describe any methods used to synthesize results and provide a rationale for the choice(s). If meta-analysis was performed, describe the model(s), method(s) to identify the presence and extent of statistical heterogeneity, and software package(s) used.	N/A
	13e	Describe any methods used to explore possible causes of heterogeneity among study results (e.g. subgroup analysis, meta-regression).	N/A
	13f	Describe any sensitivity analyses conducted to assess robustness of the synthesized results.	N/A
Study characteristics	17	Cite each included study and present its characteristics.	Table 1
Risk of bias in studies	18	Present assessments of risk of bias for each included study.	16, Appendix
Results of individual studies	19	For all outcomes, present, for each study: (a) summary statistics for each group (where appropriate) and (b) an effect estimate and its precision (e.g. confidence/credible interval), ideally using structured tables or plots.	N/A
Results of syntheses	20a	For each synthesis, briefly summarise the characteristics and risk of bias among contributing studies.	N/A
	20b	Present results of all statistical syntheses conducted. If meta-analysis was done, present for each the summary estimate and its precision (e.g. confidence/credible interval) and measures of statistical heterogeneity. If comparing groups, describe the direction of the effect.	N/A
	20c	Present results of all investigations of possible causes of heterogeneity among study results.	N/A
	20d	Present results of all sensitivity analyses conducted to assess the robustness of the synthesized results.	N/A
Reporting biases	21	Present assessments of risk of bias due to missing results (arising from reporting biases) for each synthesis assessed.	16, Appendix
Certainty of evidence	22	Present assessments of certainty (or confidence) in the body of evidence for each outcome assessed.	N/A
**DISCUSSION**			
Discussion	23a	Provide a general interpretation of the results in the context of other evidence.	17

	23b	Discuss any limitations of the evidence included in the review.	21
	23c	Discuss any limitations of the review processes used.	21
	23d	Discuss implications of the results for practice, policy, and future research.	20
**OTHER INFORMATION**			
Registration and protocol	24a	Provide registration information for the review, including register name and registration number, or state that the review was not registered.	5
	24b	Indicate where the review protocol can be accessed, or state that a protocol was not prepared.	5
	24c	Describe and explain any amendments to information provided at registration or in the protocol.	5
Support	25	Describe sources of financial or non-financial support for the review, and the role of the funders or sponsors in the review.	22
Competing interests	26	Declare any competing interests of review authors.	22
Availability of data, code and other materials	27	Report which of the following are publicly available and where they can be found: template data collection forms; data extracted from included studies; data used for all analyses; analytic code; any other materials used in the review.	22

Table S2: Search strategy

PubMed	(("Chemokines"[MeSH] OR chemokines [tiab] OR cytokines [tiab] OR “chemokine receptor” [tiab])<br>AND<br>("Biosensing Techniques"[MeSH] OR "Biomarkers"[MeSH] OR biosensors [tiab] OR biomarkers [tiab] OR sensors [tiab] OR "Biosensing Techniques"[tiab])<br>AND<br>("Prognosis"[MeSH] OR "Diagnosis"[MeSH] OR prognos* [tiab] OR diagnos* [tiab])<br>AND<br>("Head and Neck Neoplasms"[MeSH] OR "Oropharyngeal Neoplasms"[MeSH] OR "Laryngeal Neoplasms"[MeSH] OR "Head and Neck Neoplasm"[tiab] OR "Oropharyngeal Neoplasm"[tiab] OR "Laryngeal Neoplasm"[tiab] OR “head and neck cancer” [tiab] OR “head and neck squamous cell carcinoma” [tiab] OR HNSCC [tiab] OR “oral cancer” [tiab] OR “oropharyngeal cancer” [tiab] OR “laryngeal cancer” [tiab]))
Embase	((chemokines:ti,ab OR cytokines:ti,ab OR 'chemokine receptor':ti,ab)<br>AND<br>(biosensors:ti,ab OR biomarkers:ti,ab OR sensors:ti,ab OR 'Biosensing Techniques':ti,ab)<br>AND<br>(prognos*:ti,ab OR diagnos*:ti,ab)<br>AND<br>('Head and Neck Neoplasm':ti,ab OR 'Oropharyngeal Neoplasm':ti,ab OR 'Laryngeal Neoplasm':ti,ab OR 'head and neck cancer':ti,ab OR 'head and neck squamous cell carcinoma':ti,ab OR HNSCC:ti,ab OR 'oral cancer':ti,ab OR 'oropharyngeal cancer':ti,ab OR 'laryngeal cancer':ti,ab))
Scopus	((TITLE-ABS(chemokines) OR TITLE-ABS(cytokines) OR TITLE-ABS("chemokine receptor"))<br>AND<br>(TITLE-ABS(biosensors) OR TITLE-ABS(biomarkers) OR TITLE-ABS(sensors) OR TITLE-ABS("Biosensing Techniques"))<br>AND<br>(TITLE-ABS(prognos*) OR TITLE-ABS(diagnos*))<br>AND<br>(TITLE-ABS("Head and Neck Neoplasm") OR TITLE-ABS("Oropharyngeal Neoplasm") OR TITLE-ABS("Laryngeal Neoplasm") OR TITLE-ABS("head and neck cancer") OR TITLE-ABS("head and neck squamous cell carcinoma") OR TITLE-ABS(HNSCC) OR TITLE-ABS("oral cancer") OR TITLE-ABS("oropharyngeal cancer") OR TITLE-ABS("laryngeal cancer")))
Web of Science	TS=((chemokines OR cytokines OR "chemokine receptor")<br>AND<br>(biosensors OR biomarkers OR sensors OR "Biosensing Techniques")<br>AND<br>(prognos* OR diagnos*)<br>AND<br>("Head and Neck Neoplasm" OR "Oropharyngeal Neoplasm" OR "Laryngeal Neoplasm" OR "head and neck cancer" OR "head and neck squamous cell carcinoma" OR HNSCC OR "oral cancer" OR "oropharyngeal cancer" OR "laryngeal cancer"))
Ebsco	(((MH Chemokines+) OR (TI chemokines OR AB chemokines) OR (TI cytokines OR AB cytokines) OR (TI "chemokine receptor" OR AB "chemokine receptor"))<br>AND<br>((MH "Biosensing Techniques+") OR (MH Biomarkers+) OR (TI biosensors OR AB biosensors) OR (TI biomarkers OR AB biomarkers) OR (TI sensors OR AB sensors) OR (TI "Biosensing Techniques" OR AB "Biosensing Techniques"))<br>AND<br>((MH Prognosis+) OR (MH Diagnosis+) OR (TI prognos* OR AB prognos*) OR (TI diagnos* OR AB diagnos*))<br>AND<br>((MH "Head and Neck Neoplasms+") OR (MH "Oropharyngeal Neoplasms+") OR (MH "Laryngeal Neoplasms+") OR (TI "Head and Neck Neoplasm" OR AB "Head and Neck Neoplasm") OR (TI "Oropharyngeal Neoplasm" OR AB "Oropharyngeal Neoplasm") OR (TI "Laryngeal Neoplasm" OR AB "Laryngeal Neoplasm") OR (TI "head and neck cancer" OR AB "head and neck cancer") OR (TI "head and neck squamous cell carcinoma" OR AB "head and neck squamous cell carcinoma") OR (TI HNSCC OR AB HNSCC) OR (TI "oral cancer" OR AB "oral cancer") OR (TI "oropharyngeal cancer" OR AB "oropharyngeal cancer") OR (TI "laryngeal cancer" OR AB "laryngeal cancer")))

Table S3: Prognostic parameters of the remaining biomarkers reported in the included studies. [1-13]

Author	Tumor type	Technique	Sample type	Chemokine detected	HR (95%CI)
**Allen et al., 2007 [1]**	OPSCC	Immunoassay	Serum	GRO-1	CSS: 1.6 (1.2-2.2)
**Li et al., 2020 [2]**	HNSCC	mRNA	Tissue	CXCL1	OS: 1.52 (1.13-2.04)
					RFS 1.77 (0.82-3.84)
**Li et al., 2020 [2]**	HNSCC	mRNA	Tissue	CXCL2	OS: 1.24 (0.93-1.66)
					DFS: 2.49 (1.15-5.39)
**Li et al., 2020 [2]**	HNSCC	mRNA	Tissue	CXCL3	OS: 0.88 (0.66-1.17)
					DFS: 2.13 (1.02-4.48)
**Li et al., 2020** [2]	HNSCC	mRNA	Tissue	CXCL4	OS: 1.23 (0.90-1.68)
					DFS: 2.11 (0.97-4.59)
**Li et al., 2020 [2]**	HNSCC	mRNA	Tissue	CXCL5	OS: 0.83 (0.62-1.11)
					DFS: 0.56 (0.26-1.19)
**Li et al., 2020 [2]**	HNSCC	mRNA	Tissue	CXCL6	OS: 1.24 (0.95-1.63)
					DFS: 1.55 (0.73-3.27)
**Li et al., 2020 [2]**	HNSCC	mRNA	Tissue	CXCL7	OS: 1.20 (0.92-1.58)
					DFS: 0.61 (0.29-1.31)
**Li et al., 2020 [2]**	HNSCC	mRNA	Tissue	CXCL8	OS: 1.54 (1.15-2.07)
					DFS: 1.72 (0.79-3.73)
**Wang et al., 2024 [3]**	HNSCC	H&E	Tissue	CXCL8	OS: 1.52 (1.12-2.05)
**Li et al., 2020 [2]**	HNSCC	mRNA	Tissue	CXCL9	OS: 0.65 (0.49-0.87)

					DFS: 2.53 (0.96-6.67)
**Chang et al., 2013 [4]**	OSCC	ELISA	Serum	CXCL9	OS: 2.37 (1.32-4.26)
					DFS: 2.50 (1.32-4.72)
**Li et al., 2020 [2]**	HNSCC	mRNA	Tissue	CXCL11	OS: 0.76 (0.58-1.00)
					DFS: 1.99 (0.93-4.27)
**Li et al., 2020 [2]**[2]	HNSCC	mRNA	Tissue	CXCL12	OS: 0.84 (0.62-1.13)
					DFS: 2.93 (1.39-6.16)
**Li et al., 2020 [2]**	HNSCC	mRNA	Tissue	CXCL13	OS: 0.58 (0.41-0.81)
					DFS: 1.57 (0.75-3.30)
**Li et al., 2020 [2]**	HNSCC	mRNA	Tissue	CXCL14	OS: 0.74 (0.56-0.97)
					DFS: 0.47 (0.22-0.99)
**Li et al., 2020 [2]**	HNSCC	mRNA	Tissue	CXCL16	OS: 0.82 (0.61-1.09)
					DFS: 1.43 (0.63-3.26)
**Li et al., 2020 [2]**	HNSCC	mRNA	Tissue	CXCL17	OS: 0.68 (0.52-0.89)
					DFS: 0.50 (0.24-1.07)
**Wang et al., 2019 [5]**	TSCC	IHC	Tissue	CCR4	OS: 5.12 (1.82-14.82)
					DFS: 3.40 (1.40-8.23)
**Li et al., 2022 [6]**	HNSCC	RT-qPCR	Tissue	CCR5	OS: 0.59 (0.45-0.78)
**Li et al., 2022 [6]**	HNSCC	RT-qPCR	Tissue	CCR5	RFS: 3.27 (1.55-6.88)
**Domingueti et al., 2019 [7]**	OSCC	IHC	Tissue	CCR7	DFS: 3.41 (1.00 – 11.76)
**Tsuzuki et al., 2006 [8]**	OSCC/OPSCC	IHC	Tissue	CCR7	OS: 2.72 (1.05-7.06)
**Lu et al., 2011 [9]**	NPC	ELISA	Serum	CCL2	OS: 2.08 (1.28-3.38)
					DMFS: 1.92 (1.15-3.20)

**Mao et al., 2020 [10]**	OSCC	IHC	Tissue	CCL18	OS: 2.64 (1.07-6.51)
					DFS: 2.96 (1.21-7.28)
**Sun et al., 2011 [11]**	LSCC	IHC	Tissue	DARC	OS: 21.48 (1.29-355.68)
**Shen et al., 2022 [12]**	HNSCC	HTSeq FPKM	Tissue	CXCR3	OS: 0.66 (0.48-0.89)
**Shen et al., 2022 [12]**	HNSCC	HTSeq FPKM	Tissue	CXCR5	OS: 0.61 (0.45-0.82)
**Shen et al., 2022 [12]**	HNSCC	HTSeq FPKM	Tissue	CXCR6	OS: 0.71 (0.51-0.97)
**Yanagiya et al., 2021 [13]**	OSCC	IHC	Tissue	CXCR7	OS: 8.23 (1.03-65.84)
					DFS: 4.73 (1.78-12.59)

Abbreviations: CSS, cancer specific survival; OS, overall survival; DFS, disease-free survival; PFS, progression-free survival; DSS, disease-specific survival; RFS, recurrence-free survival; DMFS, distant metastasis-free survival; DSS, disease-specific survival; MFS, metastasis-free survival; LRC, loco-regional control.

Table S4: The diagnostic performance of the remaining chemokines. [14–16]

Author	Tumor type	Technique	Sample type	Chemokine detected	Sensitivity	Specificity
**Lee et al., 2018 [14]**	OSCC	MBA	Plasma	CXCL10	78.04%	70.83%
**Lee et al., 2018 [14]**	OSCC	MBA	Saliva	Eotaxin	70.73%	62.50%
**Lee et al., 2018 [14]**	OSCC	MBA	Saliva	MIP-1B	58.54%	79.17%
**Dikova et al., 2021 [15]**	OSCC	MBA	Saliva	HCC-1	83.33%	92.00%
**Dikova et al., 2021 [15]**	OSCC	MBA	Saliva	PF-4	63.64%	80.00%
**Mao et al., 2018 [16]**	NPC	ELISA	Plasma	CCL27	67.31%	73.21%

Abbreviations: C-X-C motif chemokine ligand 10 (CXCL10) eotaxin, macrophage inflammatory protein 1β (MIP-1β), HCC-1, platelet factor 4 (PF-4), and C-C motif chemokine ligand 27 (CCL27).

Table S5: Newcastle-Ottawa Scale evaluation of included prognostic studies [1–5,7–13,17–33].

Author (Year)	Selection				Comparability	Outcome			Total Score	Classification
	S1	S2	S3	S4	C1	O1	O2	O3		
Lu et al., 2011 [9]	-	*	*	*	**	*	*	*	8	Good
Mao et al., 2020 [10]	-	*	*	*	*	*	*	*	7	Good
Rentoft et al., 2014 [17]	*	*	*	*	**	*	*	-	8	Good
Schiegnitz et al., 2018 [18]	*	*	*	*	*	*	*	*	8	Good
Shen et al., 2022 [12]	-	-	*	-	*	-	-	-	2	Poor
Sun et al., 2011 [11]	*	*	*	*	*	*	*	*	8	Good
Tang et al., 2015 [19]	-	*	*	*	*	*	*	*	7	Good
Allen et al., 2007 [1]	-	-	*	-	*	*	*	-	4	Poor
Chang et al., 2011 [20]	*	*	*	-	-	*	*	-	5	Poor
Domingueti et al., 2019 [7]	*	-	*	*	**	*	*	*	8	Good
Economopoulou et al., 2019 [21]	*	*	*	*	*	*	*	-	7	Good
Fujita et al., 2014 [22]	-	*	*	-	*	*	*	*	6	Fair
Wang et al., 2019 [5]	*	*	*	*	**	*	*	*	9	Good
Wang et al., 2024 [3]	*	*	*	*	**	*	*	*	9	Good
Wang et al., 2021 [23]	*	*	*	-	-	*	*	*	6	Poor

Yanagiya et al., 2021 [13]	*	*	*	*	**	*	*	*	9	Good
Zergoun et al., 2022 [24]	*	*	*	*	**	*	*	*	9	Good
Hao et al., 2012 [25]	*	*	*	*	*	*	*	*	8	Good
Jotic et al., 2022 [26]	*	*	*	*	**	*	*	*	9	Good
Chang et al., 2008 [27]	*	*	*	*	**	*	*	*	9	Good
Chang et al., 2013 [4]	*	*	-	-	*	*	*	*	6	Fair
Cheng et al., 2014 [28]	*	-	*	*	-	*	*	*	6	Poor
Chiara De-Colle et al., 2018 [29]	*	-	*	-	*	*	*	*	6	Fair
Chiara De-Colle et al., 2017 [30]	*	-	*	-	-	*	*	*	5	Poor
Li et al., 2020 [2]	*	-	*	-	-	*	*	*	5	Good
J. Lee et al., 2009 [31]	*	-	*	*	*	*	*	*	7	Good
Huang et al., 2022 [32]	*	*	*	-	**	*	*	*	8	Good
Tao et al., 2016 [33]	*	*	*	-	**	*	*	*	8	Good
Tsuzuki et al., 2006 [8]	*	-	*	*	**	*	*	*	8	Good

Table S6: QUADAS evaluation of included diagnostic studies. [6,14–16,34–40].

**Author (Year)**	**Domain 1: Patient Selection**				**Domain 2: Index Test**			**Domain 3: Reference Standard**			**Domain 4: Flow and Timing**				**To**
	**SG**	**SG**	**SG**	**SG**	**SG**	**SG**	**SG**	**SG**	**SG**	**SG 10**	**SG 11**	**SG 12**	**SG 13**	**SG 14**	
Mao et al., 2018 [16]	1	0	1	1	0	0	1	1	1	1	0	1	1	1	10
Rajkumar et al., 2014 [34]	1	0	1	1	1	0	1	1	1	1	0	1	1	1	11
Singh et al., 2020 [35]	1	0	1	0	0	1	1	1	0	1	1	1	1	1	10
St John et al., 2004 [36]	1	0	1	0	0	1	1	1	0	1	1	1	1	1	10
Susha and Ravindran, 2023 [37]	1	0	1	0	0	1	1	1	0	1	1	1	1	1	11
Dikova et al., 2021 [15]	1	0	1	0	0	1	1	1	0	1	0	1	1	1	9
Ghallab and Shaker, 2017 [38]	1	0	1	0	0	1	1	1	0	1	1	1	1	1	10

Gleber-Netto et al., 2016 [39]	1	0	1	0	0	0	1	1	0	1	1	1	1	1	9
Hamad et al., 2011 [40]	1	0	1	0	0	0	1	1	0	1	1	1	1	1	9
Lee et al., 2018 [14]	1	1	1	1	0	1	0	1	0	0	1	1	1	1	10
Li et al. 2022 [6]	1	0	1	1	1	1	1	1	0	0	0	1	0	0	8

References

1. Allen, C.T.; Duffy, S.; Teknos, T.N.; Islam, M.; Chen, Z.; Albert, P.S.; Wolf, G.T.; Van Waes, C. Nuclear Factor-κB–Related Serum Factors as Longitudinal Biomarkers of Response and Survival in Advanced Oropharyngeal Carcinoma. *Clin. Cancer Res.* **2007**, *13*, 3182–3190. https://doi.org/10.1158/1078-0432.ccr-06-3047.
2. Li, Y.; Wu, T.; Gong, S.; Zhou, H.; Yu, L.; Liang, M.; Shi, R.; Wu, Z.; Zhang, J.; Li, S. Analysis of the Prognosis and Therapeutic Value of the CXC Chemokine Family in Head and Neck Squamous Cell Carcinoma. *Front. Oncol.* **2021**, *10*, 570736. https://doi.org/10.3389/fonc.2020.570736.
3. Wang, W.; Ruan, S.; Xie, Y.; Fang, S.; Yang, J.; Li, X.; Zhang, Y. Development and Validation of a Pathomics Model Using Machine Learning to Predict CXCL8 Expression and Prognosis in Head and Neck Cancer. *Clin. Exp. Otorhinolaryngol.* **2024**, *17*, 85–97. https://doi.org/10.21053/ceo.2023.00026.
4. Chang, K.-P.; Wu, C.-C.; Fang, K.-H.; Tsai, C.-Y.; Chang, Y.-L.; Liu, S.-C.; Kao, H.-K. Serum levels of chemokine (C-X-C motif) ligand 9 (CXCL9) are associated with tumor progression and treatment outcome in patients with oral cavity squamous cell carcinoma. *Oral Oncol.* **2013**, *49*, 802–807. https://doi.org/10.1016/j.oraloncology.2013.05.006.
5. Wang, L.; Zhang, M.; Zhu, Y.; Zhang, X.; Yang, Y.; Wang, C. CCR4 Expression Is Associated With Poor Prognosis in Patients With Early Stage (pN0) Oral Tongue Cancer. *J. Oral Maxillofac. Surg.* **2019**, *77*, 426–432. https://doi.org/10.1016/j.joms.2018.09.035.
6. Li, C.; Chen, S.; Liu, C.; Mo, C.; Gong, W.; Hu, J.; He, M.; Xie, L.; Hou, X.; Tang, J.; et al. CCR5 as a prognostic biomarker correlated with immune infiltrates in head and neck squamous cell carcinoma by bioinformatic study. *Hereditas* **2022**, *159*, 37. https://doi.org/10.1186/s41065-022-00251-y.
7. Domingueti, C.B.; Janini, J.-B.-M.; Paranaiba, L.M.; Lozano-Burgos, C.; Olivero, P.; Gonzalez-Arriagada, W.A. Prognostic value of immunoexpression of CCR4, CCR5, CCR7 and CXCR4 in squamous cell carcinoma of tongue

and floor of the mouth. Med. Oral Patol. Oral Cir. Bucal **2019**, *24*, e354–e363. https://doi.org/10.4317/medoral.22904.
8. Tsuzuki, H.; Takahashi, N.; Kojima, A.; Narita, N.; Sunaga, H.; Takabayashi, T.; Fujieda, S. Oral and oropharyngeal squamous cell carcinomas expressing CCR7 have poor prognoses. *Auris Nasus Larynx* **2006**, *33*, 37–42. https://doi.org/10.1016/j.anl.2005.07.019.
9. Lu, X.; Qian, C.-N.; Mu, Y.-G.; Li, N.-W.; Li, S.; Zhang, H.-B.; Li, S.-W.; Wang, F.-L.; Guo, X.; Xiang, Y.-Q. Serum CCL2 and serum TNF-α – Two new biomarkers predict bone invasion, post-treatment distant metastasis and poor overall survival in nasopharyngeal carcinoma. *Eur. J. Cancer* **2011**, *47*, 339–346. https://doi.org/10.1016/j.ejca.2010.09.025.
10. Mao, L.; Zhuang, R.; Qin, L.; Han, Z.; Huang, X.; Chen, R.; Su, Y.; Ge, L.; Yang, J.; Li, J.; et al. CCL18 overexpression predicts a worse prognosis in oral squamous cell carcinoma (OSCC). *Neoplasma* **2020**, *67*, 700–706. https://doi.org/10.4149/neo_2020_190821n802.
11. Sun, G.; Wang, Y.; Zhu, Y.; Huang, C.; Ji, Q. Duffy antigen receptor for chemokines in laryngeal squamous cell carcinoma as a negative regulator. *Acta Oto-Laryngologica* **2010**, *131*, 197–203. https://doi.org/10.3109/00016489.2010.516012.
12. Shen, Y.; Zhou, C.; Cao, Y.; Li, Q.; Deng, H.; Gu, S.; Wu, Y.; Shen, Z. Expression profile and prognostic value of CXCR family members in head and neck squamous cell carcinoma. *World J. Surg. Oncol.* **2022**, *20*, 259. https://doi.org/10.1186/s12957-022-02713-z.
13. Yanagiya, M.; Dawood, R.I.H.; Maishi, N.; Hida, Y.; Torii, C.; Annan, D.A.; Kikuchi, H.; Matsuda, A.Y.; Kitamura, T.; Ohiro, Y.; et al. Correlation between endothelial CXCR7 expression and clinicopathological factors in oral squamous cell carcinoma. *Pathol. Int.* **2021**, *71*, 383–391. https://doi.org/10.1111/pin.13094.
14. Lee, L.; Wong, Y.; Hsiao, H.; Wang, Y.; Chan, M.; Chang, K. Evaluation of saliva and plasma cytokine biomarkers in patients with oral squamous cell carcinoma. *Int. J. Oral Maxillofac. Surg.* **2018**, *47*, 699–707. https://doi.org/10.1016/j.ijom.2017.09.016.
15. Dikova, V.; Jantus-Lewintre, E.; Bagan, J. Potential Non-Invasive Biomarkers for Early Diagnosis of Oral Squamous Cell Carcinoma. *J. Clin. Med.* **2021**, *10*, 1658. https://doi.org/10.3390/jcm10081658.
16. Mao, M.-J.; Xue, N.; Wang, X.-P.; Chi, P.-D.; Liu, Y.-J.; Huang, Q.; Dai, S.-Q.; Liu, W.-L. Chemokine CCL27 is a novel plasma biomarker for identification the nasopharyngeal carcinoma patients from the Epstein-Barr virus capsid antigen-specific IgA seropositive population. *BMC Cancer* **2018**, *18*, 9. https://doi.org/10.1186/s12885-017-3718-2.
17. Rentoft, M.; Coates, P.J.; Loljung, L.; Wilms, T.; Laurell, G.; Nylander, K. Expression of CXCL10 is associated with response to radiotherapy and overall survival in squamous cell carcinoma of the tongue. *Tumor Biol.* **2014**, *35*, 4191–4198. https://doi.org/10.1007/s13277-013-1549-6.
18. Schiegnitz, E.; Kämmerer, P.W.; Schön, H.; Blatt, S.; Berres, M.; Sagheb, K.; Al-Nawas, B. Proinflammatory cytokines as serum biomarker in oral carcinoma—A prospective multi-biomarker approach. *J. Oral Pathol. Med.* **2017**, *47*, 268–274. https://doi.org/10.1111/jop.12670.
19. Tang, M.; Ou, N.; Li, C.; Lu, A.; Li, J.; Ma, L.; Zhong, W.; Gao, J.; Zheng, Y.; Cai, Y. Expression and Prognostic Significance of Macrophage Inflammatory Protein-3 Alpha and Cystatin A in Nasopharyngeal Carcinoma. *BioMed Res. Int.* **2015**, *2015*, 617143. https://doi.org/10.1155/2015/617143.
20. Chang, K.; Chang, Y.; Wu, C.; Liu, Y.; Chen, M.; Tsang, N.; Hsu, C.; Chang, Y.; Yu, J. Multiplexed immunobead-based profiling of cytokine markers for detection of nasopharyngeal carcinoma and prognosis of patient survival. *Head Neck* **2010**, *33*, 886–897. https://doi.org/10.1002/hed.21557.
21. Economopoulou, P.; Koutsodontis, G.; Strati, A.; Kirodimos, E.; Giotakis, E.; Maragoudakis, P.; Prikas, C.; Papadimitriou, N.; Perisanidis, C.; Gagari, E.; et al. Surrogates of immunologic cell death (ICD) and chemoradiotherapy outcomes in head and neck squamous cell carcinoma (HNSCC). *Oral Oncol.* **2019**, *94*, 93–100. https://doi.org/10.1016/j.oraloncology.2019.05.019.

22. Fujita, Y.; Okamoto, M.; Goda, H.; Tano, T.; Nakashiro, K.-I.; Sugita, A.; Fujita, T.; Koido, S.; Homma, S.; Kawakami, Y.; et al. Prognostic Significance of Interleukin-8 and CD163-Positive Cell-Infiltration in Tumor Tissues in Patients with Oral Squamous Cell Carcinoma. *PLOS ONE* **2014**, *9*, e110378. https://doi.org/10.1371/journal.pone.0110378.
23. Wang, Y.; Li, M.; Guo, Y.; Huang, H.; Dong, X.; Sun, Y.; Liu, J. Key genes affecting the progression of nasopharyngeal carcinoma identified by RNA-sequencing and bioinformatic analysis. *Aging* **2021**, *13*, 22176–22187. https://doi.org/10.18632/aging.203521.
24. Zergoun, A.A.; Draleau, K.S.; Chettibi, F.; Touil-Boukoffa, C.; Djennaoui, D.; Merghoub, T.; Bourouba, M. Plasma secretome analyses identify IL-8 and nitrites as predictors of poor prognosis in nasopharyngeal carcinoma patients. *Cytokine* **2022**, *153*, 155852–155852. https://doi.org/10.1016/j.cyto.2022.155852.
25. Hao, W.; Zhu, Y.; Zhou, H. Prognostic value of interleukin-6 and interleukin-8 in laryngeal squamous cell cancer. *Med. Oncol.* **2013**, *30*, 333. https://doi.org/10.1007/s12032-012-0333-6.
26. Jotic, A.; Milovanovic, J.; Savic-Vujovic, K.; Radin, Z.; Medic, B.; Folic, M.; Pavlovic, B.; Vujovic, A.; Dundjerovic, D. Immune Cell and Biochemical Biomarkers in Advanced Laryngeal Cancer. *Dose-Response* **2022**, *20*, 1–9. https://doi.org/10.1177/15593258221115537.
27. Chang, K.-P.; Hao, S.-P.; Chang, J.-H.; Wu, C.-C.; Tsang, N.-M.; Lee, Y.-S.; Hsu, C.-L.; Ueng, S.-H.; Liu, S.-C.; Liu, Y.-L.; et al. Macrophage Inflammatory Protein-3α Is a Novel Serum Marker for Nasopharyngeal Carcinoma Detection and Prediction of Treatment Outcomes. *Clin. Cancer Res.* **2008**, *14*, 6979–6987. https://doi.org/10.1158/1078-0432.ccr-08-0090.
28. Cheng, D.; Kong, H.; Li, Y. Prognostic value of interleukin-8 and MMP-9 in nasopharyngeal carcinoma. *Eur. Arch. Oto-Rhino-Laryngology* **2013**, *271*, 503–509. https://doi.org/10.1007/s00405-013-2580-3.
29. De-Colle, C.; Menegakis, A.; Mönnich, D.; Welz, S.; Boeke, S.; Sipos, B.; Fend, F.; Mauz, P.-S.; Tinhofer, I.; Volker Budach, V.; Jawad, J.A.; et al. SDF-1/CXCR4 expression is an independent negative prognostic biomarker in patients with head and neck cancer after primary radiochemotherapy. *Radiother Oncol.* **2018**, *126*, 125-131. https://doi:10.1016/j.radonc.2017.10.008.
30. De-Colle, C.; Mönnich, D.; Welz, S.; Boeke, S.; Sipos, B.; Fend, F.; Mauz, P.-S.; Tinhofer, I.; Budach, V.; Abu Jawad, J.; et al. SDF-1/CXCR4 expression in head and neck cancer and outcome after postoperative radiochemotherapy. *Clin. Transl. Radiat. Oncol.* **2017**, *5*, 28–36. https://doi.org/10.1016/j.ctro.2017.06.004.
31. Lee, J.-I.; Jin, B.-H.; Kim, M.-A.; Yoon, H.-J.; Hong, S.-P.; Hong, S.-D. Prognostic significance of CXCR-4 expression in oral squamous cell carcinoma. *Oral Surgery, Oral Med. Oral Pathol. Oral Radiol. Endodontology* **2009**, *107*, 678–684. https://doi.org/10.1016/j.tripleo.2008.12.047.
32. Huang, Y.-C.; Huang, J.-L.; Tseng, L.-C.; Yu, P.-H.; Chen, S.-Y.; Lin, C.-S. High Expression of Interferon Pathway Genes CXCL10 and STAT2 Is Associated with Activated T-Cell Signature and Better Outcome of Oral Cancer Patients. *J. Pers. Med.* **2022**, *12*, 140. https://doi.org/10.3390/jpm12020140.
33. Tao, H.; Wei, Y.; Wang, C.; Yang, K.; Huang, W.; Liu, H.; Li, B. Expression of chemokine receptor CXCR4 is closely correlated with clinical outcome in human nasopharyngeal carcinoma. *Tumor Biol.* **2015**, *37*, 6099–6105. https://doi.org/10.1007/s13277-015-4464-1.
34. Rajkumar, K.; Nandhini, G.; Ramya, R.; Rajashree, P.; Kumar, A.R.; Anandan, S.N. Validation of the diagnostic utility of salivary interleukin 8 in the differentiation of potentially malignant oral lesions and oral squamous cell carcinoma in a region with high endemicity. *Oral Surgery, Oral Med. Oral Pathol. Oral Radiol.* **2014**, *118*, 309–319. https://doi.org/10.1016/j.oooo.2014.04.008.
35. Singh, P.; Verma, J.K.; Singh, J.K. Validation of Salivary Markers, IL-1β, IL-8 and Lgals3bp for Detection of Oral Squamous Cell Carcinoma in an Indian Population. *Sci. Rep.* **2020**, *10*, 7365. https://doi.org/10.1038/s41598-020-64494-3.

36. John, M.A.R.S.; Li, Y.; Zhou, X.; Denny, P.; Ho, C.-M.; Montemagno, C.; Shi, W.; Qi, F.; Wu, B.; Sinha, U.; et al. Interleukin 6 and Interleukin 8 as Potential Biomarkers for Oral Cavity and Oropharyngeal Squamous Cell Carcinoma. *Arch. Otolaryngol. Neck Surg.* **2004**, *130*, 929–935. https://doi.org/10.1001/archotol.130.8.929.
37. Susha, K.P.; Ravindran, R. Evaluation of salivary Chemerin in oral leukoplakia, oral squamous cell carcinoma and healthy controls. *J. Orofac. Sci.* **2023**, *15*, 141–146. https://doi.org/10.4103/jofs.jofs_40_23.
38. Ghallab, N.A.; Shaker, O.G. Serum and salivary levels of chemerin and MMP-9 in oral squamous cell carcinoma and oral premalignant lesions. *Clin. Oral Investig.* **2016**, *21*, 937–947. https://doi.org/10.1007/s00784-016-1846-8.
39. Gleber-Netto, F.O.; Yakob, M.; Li, F.; Feng, Z.; Dai, J.; Kao, H.-K.; Chang, Y.-L.; Chang, K.-P.; Wong, D.T. Salivary Biomarkers for Detection of Oral Squamous Cell Carcinoma in a Taiwanese Population. *Clin. Cancer Res.* **2016**, *22*, 3340–3347. https://doi.org/10.1158/1078-0432.ccr-15-1761.
40. Hamad AWR, Gaphor SM, Shawagfeh MT, Al-Talabani NG. Study of serum and salivary levels of proinflammatory cytokines, potential biomarkers in the diagnosis of oral squamous cell carcinoma. Academic Journal of Cancer Research. **2011**, 4(2):47-55.
